# The Conformation of Glycosidic Linkages According to Various Force Fields: Monte Carlo Modeling of Polysaccharides Based on Extrapolation of Short-Chain Properties

**DOI:** 10.1021/acs.jctc.4c00543

**Published:** 2024-07-10

**Authors:** Valery Lutsyk, Pawel Wolski, Wojciech Plazinski

**Affiliations:** † Jerzy Haber Institute of Catalysis and Surface Chemistry, Polish Academy of Sciences, Niezapominajek 8, 30-239 Krakow, Poland; ‡ Department of Biopharmacy, Medical University of Lublin, Chodzki 4a, 20-093 Lublin, Poland

## Abstract

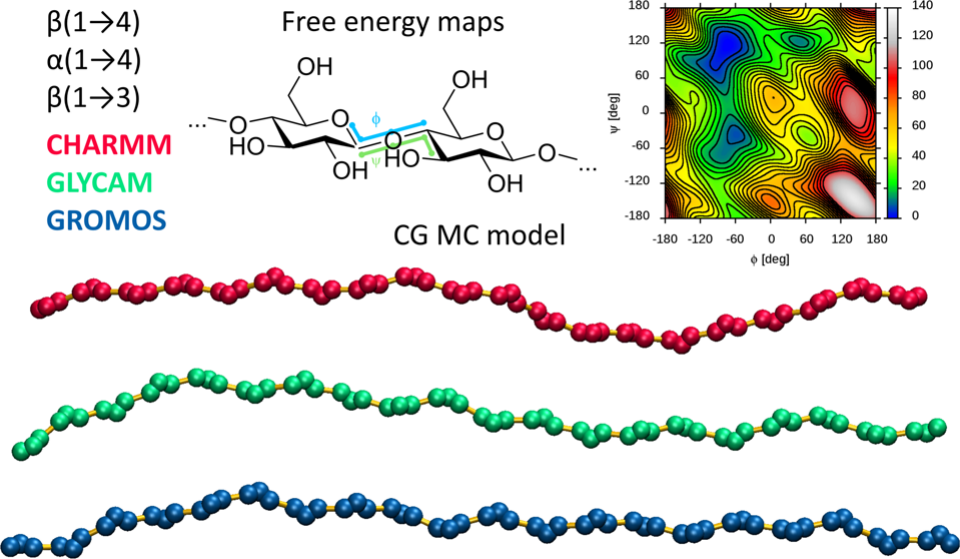

The conformational features of the glycosidic linkage are the most important variable to consider when studying di-, oligo-, and polysaccharide molecules using molecular dynamics (MD) simulations. The accuracy of the theoretical model describing this degree of freedom influences the quality of the results obtained from MD calculations based on this model. This article focuses on the following two issues related to the conformation of the glycosidic linkage. First, we describe the results of a comparative analysis of the predictions of three carbohydrate-dedicated classical force fields for MD simulations, namely, CHARMM, GLYCAM, and GROMOS, in the context of different parameters of structural and energetic nature related to the conformation of selected types of glycosidic linkages, α(1 → 4), β(1 → 3), and β(1 → 4), connecting glucopyranose units. This analysis revealed several differences, mainly concerning the energy levels of the secondary and tertiary conformers and the linkage flexibility within the dominant *exo*-*syn* conformation for α(1 → 4) and β(1 → 3) linkages. Some aspects of the comparative analysis also included the newly developed, carbohydrate-dedicated Martini 3 coarse-grained force field. Second, to overcome the time-scale problem associated with sampling slow degrees of freedom in polysaccharide chains during MD simulations, we developed a coarse-grained (CG) model based on the data from MD simulations and designed for Monte Carlo modeling. This model (CG MC) is based on information from simulations of short saccharide chains, effectively sampled in atomistic MD simulations, and is capable of extrapolating local conformational properties to the case of polysaccharides of arbitrary length. The CG MC model has the potential to estimate the conformations of very long polysaccharide chains, taking into account the influence of secondary and tertiary conformations of glycosidic linkages. With respect to the comparative analysis of force fields, the application of CG MC modeling showed that relatively small differences in the predictions of individual force fields with respect to a single glycosidic linkage accumulate when considering their effect on the structure of longer chains, leading to drastically different predictions with respect to parameters describing the polymer conformation, such as the persistence length.

## Introduction

The conformational variability of carbohydrate molecules depends on several degrees of freedom, including the shape of the ring and the orientation of exocyclic substituents.^1^ Unless one is restricted to the simplest case of monosaccharides, the primary variable to be considered in this context is also the conformation of the glycosidic linkages, which consist of two or three covalent bonds linking adjacent monosaccharides.^2^ Glycosidic linkages can differ both in the point of attachment to the saccharide ring and in the orientation relative to the rings involved. Furthermore, in cases where a single monosaccharide has multiple linkages, the oligo- or polysaccharide chain becomes branched.

Computational methods are now a routine and widely used tool for studying the structure and conformation of carbohydrates and biomolecular systems containing carbohydrates.^3−8^ In the context of molecular dynamics (MD) simulations of carbohydrates, two key issues need to be highlighted as they are the main sources of uncertainty regarding the accuracy of MD simulation results.

The first issue concerns the accuracy of the force fields used to calculate intra- and intermolecular interactions in the system.^9−13^ While all carbohydrate-dedicated force fields provide similar predictions regarding the location of the dominant glycosidic linkage conformation (as validated by either experimental NMR data, quantum mechanical calculations, or both),^14−16^ other details of the conformational properties (e.g., the nature and relative populations of secondary conformers) may vary. In addition, even small differences in predictions for a single linkage can accumulate when considering larger, more complex systems containing carbohydrate molecules with multiple glycosidic linkages. The accuracy of the force field with respect to secondary and tertiary conformations is important because, unlike proteins, the biological functions of active carbohydrates do not depend solely on a single, basic conformation but rather on the dynamic equilibrium between multiple conformational states.^4^

The second issue concerns the time scale of the processes under study, which is closely related to the size of the simulated system and computational efficiency. Some conformational changes in saccharide molecules occur on large time scales, e.g., microseconds (ring distortions)^17^ or tens to hundreds of nanoseconds (reorientations of glycosidic linkages).^18^ Furthermore, due to the high conformational variability and hydrophilicity of saccharides, the sizes of the simulation boxes generally need to be much larger than those for proteins with a comparable number of building blocks. For example, a 40-unit cellulose polymer chain, taking into account its extended rodlike conformation, requires a cubic box size of about 21 × 21 × 21 nm^3^, corresponding to about 300,000 water molecules and a contribution of saccharide atoms in the system of only 0.1%. Obviously, this has a significant impact on computational efficiency, making it difficult to achieve convergence of results within an accessible time and often making it impossible to study the influence of slow degrees of freedom on the conformation of the entire saccharide molecule.

Both issues are addressed in this article.

In the context of a comparative analysis of force fields, we investigated the differences between the predictions of the three classical, carbohydrate-dedicated force fields with respect to the dynamic conformation of glycosidic linkages and related parameters. This aspect extends our previous works, where we investigated how the choice of force field affects the predicted values of the pyranose ring distortion energy^19^ and the CH–π interaction-driven binding between the protein and unfunctionalized carbohydrates.^12^ Related studies focusing on specific carbohydrate systems are also known.^10,20−23^ The present work aims to compare the predictions of the three biomolecular force fields most commonly used in MD simulations to study saccharide conformations, namely, CHARMM,^14,24^ GLYCAM,^15^ and GROMOS.^16,25^ The systems studied included three types of glycosidic linkages most commonly found in natural polysaccharides, namely, α(1 → 4), β(1 → 3), and β(1 → 4). The comparative analysis included various parameters of both structural and energetic nature. In addition, for selected conformational descriptors, we extended the comparative analysis by including predictions of the coarse-grained force field of the Martini family (version 3), recently developed by our team and used for molecular dynamics simulations of saccharides.^26^

In the context of time-scale issues, a coarse-grained (CG) model based on two-dimensional free-energy maps of glycosidic dihedral angles ϕ vs ψ and Monte Carlo (MC) methodology has been proposed. The model allows the rapid generation of saccharide backbone configurations with correct thermodynamic averages with respect to several polymer properties, such as end-to-end distance, radius of gyration, contour length, etc. Furthermore, the proposed CG MC model has been used to demonstrate how small differences in structural and energetic properties related to rotation around ϕ and ψ linkages can propagate and significantly affect parameters characterizing large polysaccharide molecules (e.g., persistence length).

## Methods

### Molecular Dynamics Simulations

**Figure 1:**
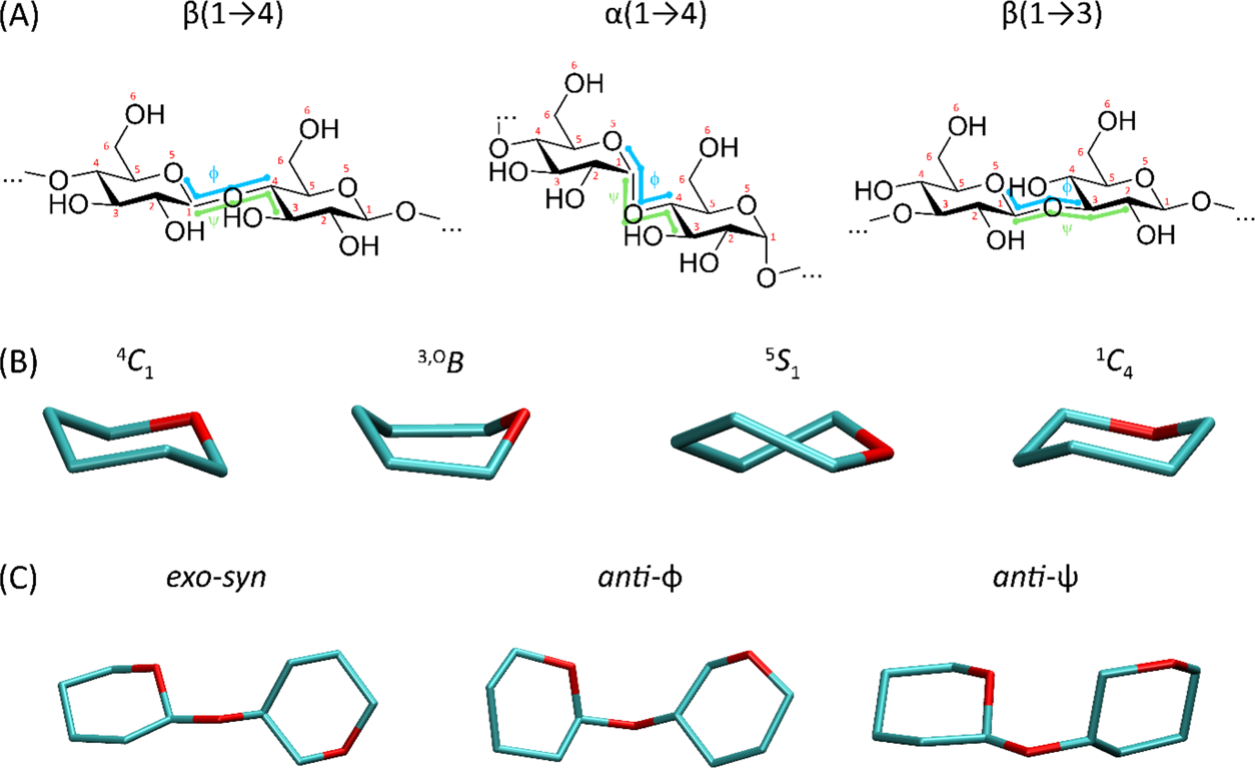
(A) The chemical formulas of the compounds studied (only disaccharide fragments are shown for clarity). The atom numbering and the definition of the glycosidic torsion angles ϕ and ψ are also shown. (B) Graphical representation of the basic conformations of the pyranose ring: regular chair (^4^*C*_1_), boat (^3,O^*B*), skew boat (^5^*S*_1_), and inverted chair (^1^*C*_4_). (C) Graphical representation of the most common conformers of the glycosidic linkage using the example of the β(1 → 4) linkage: *exo*-*syn*, *anti*-ϕ, and *anti*-ψ. In panels (B) and (C), all ring substituents have been omitted for clarity.

The MD simulations concerned the homooctamers of d-glucopyranose residues linked by the three types of glycosidic linkages: α(1 → 4), β(1 → 3), and β(1 → 4). The chemical formulas of the compounds studied are shown in Figure 1. The anomeric configuration of the reducing end was set to be the same as that of the glycosidic linkage.

Three different carbohydrate-dedicated force fields were used: (1) CHARMM36,^14,24^ (2) GLYCAM06,^15^ and (3) GROMOS 56a6_CARBO_R_.^16,25^ The force field parameters as well as the initial configurations were generated either by using the GROMACS^27^*pdb2gmx* routine based on our previous works^25,28^ (in the case of GROMOS) or by using the online server www.charmm-gui.org(29,30) (in the case of CHARMM and GLYCAM).

All MD simulations were performed with the GROMACS (2022.5 and 2023.2 versions) package.^27^ The oligomer molecules were placed in cubic simulation boxes with dimensions varying between 5.1 × 5.1 × 5.1 and 5.4 × 5.4 × 5.4 nm^3^ and surrounded by the number of explicit water molecules corresponding approximately to the system density of 1 g/cm^3^, i.e., about 4300–5050. The unbiased MD simulations were performed under periodic boundary conditions and in the isothermal–isobaric ensemble. The temperature was kept close to its reference value (298 K) using the V-rescale thermostat,^31^ while for the constant pressure (1 bar, isotropic coordinate scaling), the Parrinello–Rahman barostat^32^ was used with a relaxation time of 0.4 ps. The equations of motion were integrated with a time step of 2 fs using the leapfrog scheme.^33^ The translational center-of-mass motion was removed at each time step separately for the solute and the solvent. Full rigidity of the water molecules was enforced using the SETTLE procedure.^34^ All systems were preoptimized during an equilibration protocol lasting 1 ns of NPT simulation. After equilibration, production simulations were performed for 100 ns according to slightly different schemes described below. In the case of the β(1 → 3)Glc octamer simulated in GLYCAM, the MD simulations were extended to 1.1 μs to account for the non-negligible effects related to ring flexibility. The small differences reflect the conditions compatible with the given force field and usually correspond to the recommended setup used in the parameterization procedure. Data were saved every 1 ps.

#### CHARMM

The TIP3P model of water^35^ was used. Hydrogen-containing solute bond lengths were constrained using the LINCS procedure with a relative geometric tolerance of 10^–4^.^36^ Electrostatic interactions were modeled using the particle-mesh Ewald method^37^ with a cutoff of 1.2 nm, while van der Waals interactions (LJ potentials) were switched off between 1.0 and 1.2 nm.

#### GLYCAM

The TIP3P model of water^35^ was used. Hydrogen-containing solute bond lengths were constrained using the LINCS procedure with a relative geometric tolerance of 10^–4^.^36^ Electrostatic interactions were modeled using the particle-mesh Ewald method^37^ with a cutoff of 1 nm, while van der Waals interactions (LJ potentials) were switched off between 1.0 and 1.1 nm.

#### GROMOS

The SPC model of water^38^ was used. The solute bond lengths were constrained using the LINCS procedure with a relative geometric tolerance of 10^–4^.^36^ The nonbonded interactions were calculated using a single cutoff distance set to 1.4 nm and the Verlet list scheme. The reaction-field correction was applied to account for the average effect of electrostatic interactions beyond the long-range cutoff distance, using a relative dielectric permittivity of 61 as appropriate for the SPC water model.^39^

The enhanced-sampling free-energy calculations focused on the two-dimensional free-energy maps (2D FEMs) associated with the glycosidic linkage conformation. The variables describing the conformation of the glycosidic linkages were ϕ and ψ torsion angles defined by the following quadruplets of atoms: ϕ = O_5_–C_1_–O_1_–C_*n*_; ψ = C_1_–O_1_–C_*n*_–C_*n*+1_ (*n* = 3 or 4; see Figure 1 for details). The calculations were based on an enhanced-sampling scheme^40^ combining parallel tempering^41^ and well-tempered metadynamics,^42^ as implemented in the PLUMED 2.6 plug-in.^43^ The well-tempered metadynamics was based on local Gaussian functions with a width of 18°, an initial deposition rate of 0.01 kJ mol^–1^·ps^–1^, and a temperature parameter Δ*T* (defined according to eq 2 of Barducci et al.^42^) of 1788 K. The parallel tempering was based on 16 metadynamics simulations performed in parallel at different temperatures ranging from 298.0 to 363.2 K in steps of about 4.3 K, together with replica-exchange attempts performed at 2 ps intervals. The duration of the metadynamics simulations varied between 100 and 105 ns, depending on the time required to obtain convergent results. The remaining details of the simulation setup were identical to those described above for the case of unbiased MD simulations. Convergence was monitored using handwritten scripts and the PLUMED *sum_hills* module by checking the relative free-energy difference between the main and secondary minima. The 2D free-energy maps used for the final analyses were averaged over the last 20–36 ns of the simulations, i.e., over the range of approximately constant energy differences between the free-energy minima.

The conformations of the pyranose rings, when considered, were assigned to one of three possibilities: regular chair (^4^*C*_1_), boat/skew-boat ensemble of conformers (*B*/*S*), and inverted chair (^1^*C*_4_). The assignments were based on the values of the Cremer–Pople parameter θ and their membership in the intervals: (0; 60°), (60; 120°), and (120; 180°), respectively. The most common conformers of the glycosidic linkage were described by the terms *exo*-*syn*, *anti*-φ, and *anti*-ψ. Although more detailed definitions of these terms exist, for simplicity, we refer to them based on the location of the minima on the 2D free-energy map (FEM). The main minimum corresponds to the *exo*-*syn* conformer, while secondary/tertiary minima that have approximately the same ψ or φ coordinate value as the main minimum correspond to *anti*-φ and *anti*-ψ conformations, respectively. See also Figure 1 for a graphical illustration.

The MD simulations within the carbohydrate-dedicated coarse-grained Martini 3 force field were performed according to the methodology described in the original paper.^26^ The new simulations included chains of α(1 → 4), β(1 → 3), and β(1 → 4) glucan of 50 residues in length. The simulation length varied from 2.8 μs (curdlan) to 5 μs (cellulose and amylose). In addition, data from ref (26) were also used, especially in the context of short octameric chains.

### Simplified One-Dimensional (1D) Model for Monte Carlo Simulations

In order to investigate the influence of conformational energy levels on the configuration of glycosidic linkage conformational states within a polysaccharide chain and to determine whether such a simplified description can capture at least some of the conformational properties of real systems, a simple one-dimensional polysaccharide model has been proposed. This model is essentially identical to the general model of any polymer composed of units connected by linkages capable of assuming more than one conformational state.

The main assumptions of the model are as follows:

1. Treatment of the polysaccharide chain as a one-dimensional list whose length is identical to the number of glycosidic linkages within this chain, each position on the list corresponding to a specific conformational state.
2. The conformational states are discrete, and their number is arbitrary.
3. Each conformational state has an associated energy.
4. Glycosidic linkages (here: positions on the 1D list) are equivalent, i.e., each position corresponds to the same number of conformational states with the same energies.
5. The initial configuration contains the same conformational state for each position, corresponding to the lowest energy.

The Monte Carlo simulation procedure includes the following random steps: (1) randomly selecting a position from the 1D list and (2) attempting to change the conformational state by randomly selecting a new configuration type. The new configuration is accepted or rejected by comparing the energy differences before and after the conformational change and applying the Metropolis criterion.^44^

**Figure 2:**
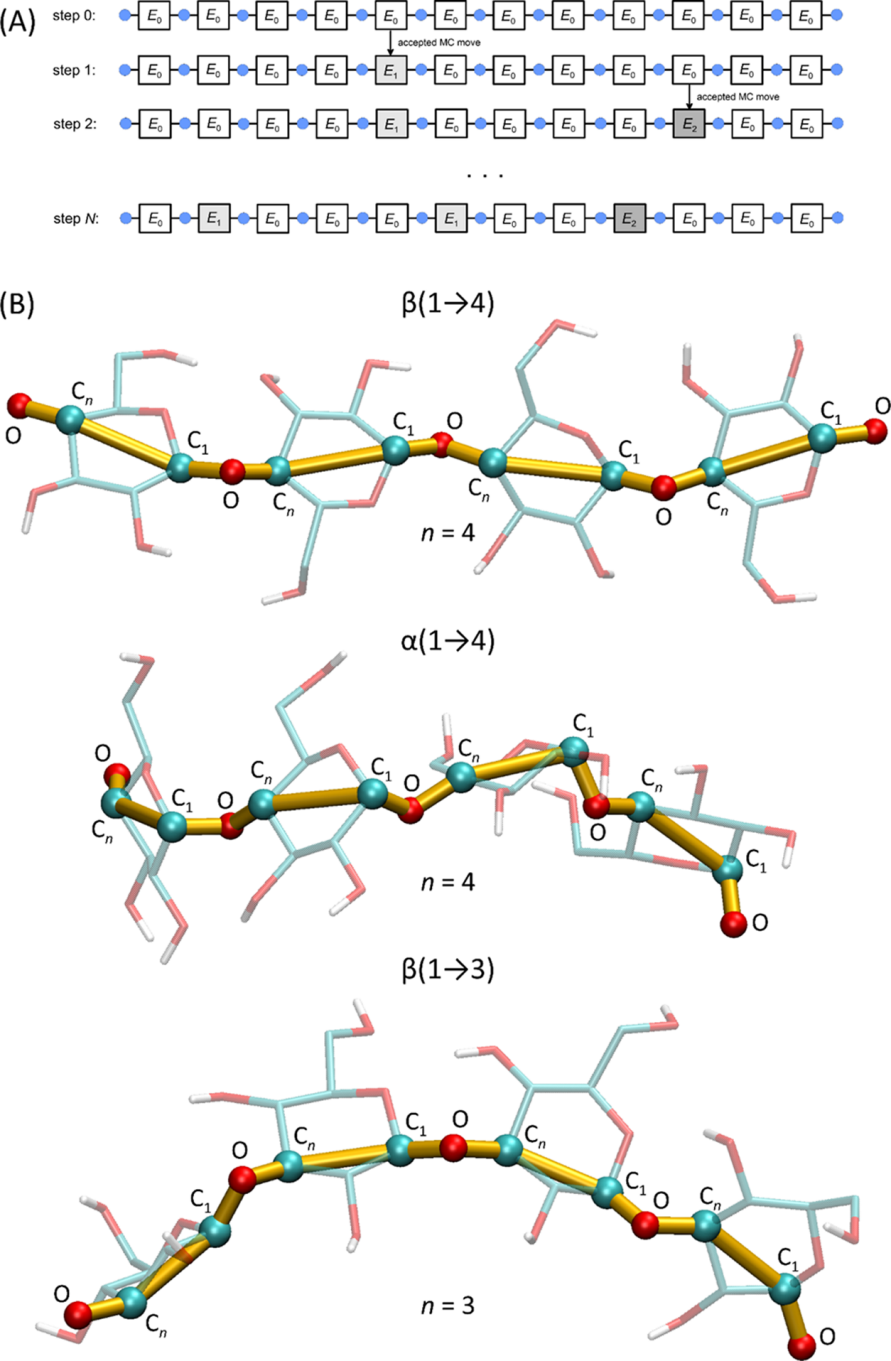
(A) Schematic representation of the 1D Monte Carlo model for a chain containing 13 units (blue circles) and 12 glycosidic linkages (squares). The initial configuration includes all linkages with the lowest energy (*E*_0_); during the simulation, new configurations (with energy increments of *E*_1_ and *E*_2_) are accepted or rejected according to the Metropolis criterion. After *N* steps, the generated configuration corresponds to the parameter value *L* = 2.25. (B) Schematic representation of the proposed coarse-grained Monte Carlo (CG MC) model. The polysaccharide chain fragments with different linkage topologies ((1 → 4) or (1 → 3)) are shown in stick representation (transparent), while atoms included in the CG representation are marked as spheres. The polysaccharide backbone, formed by the −C_*n*_–C_1_–O– repeating motif, is shown in yellow. The aliphatic hydrogen atoms have been omitted for clarity.

The main output of Monte Carlo simulations performed within such a model is the parameter *L*, defined as the average uninterrupted length of the chain fragment having the same lowest-energy conformational state. Assuming that only conformational changes between separate energy minima affect the conformation of the chain and that the contribution of conformational changes within a single energy minimum is negligible, this parameter can be treated as an approximation of the persistence length. In other words, a fragment of length *L* in which all linkages have the same low-energy conformation corresponds to a rigid rodlike geometry. Alternative higher-energy conformations of the linkages separating longer fragments of length *L* correspond to kinks in the rigid chain. To generate any configuration, both the number of conformational states per glycosidic linkage and the corresponding conformational energies must be known from the independent MD simulations. A graphical representation of the 1D Monte Carlo model is shown in Figure 2.

The simulations covered a range of chains from 500 to 5000 residues in length. The number of conformational states was set to either 2 or 3, and their energies were equal to (1) the energy of the global minimum on the free-energy maps; (2) the energies of the *anti*-ϕ and *anti*-ψ states on the same maps. The input data (energies of the conformational states) are given in Table 1.

**Table 1:** Conformational Properties of Glycosidic Linkages as Predicted by Different Force Fields^a^

		conformational properties							
		main minimum		*anti*-φ			*anti*-ψ		
force field	linkage	φ [deg]	ψ [deg]	φ [deg]	ψ [deg]	Δ*E* [kJ/mol]	φ [deg]	ψ [deg]	Δ*E* [kJ/mol]
CHARMM	β(1 → 4)	–74	117	50	123	11.5	–59	–36	7.4
	α(1 → 4)	97	99	–38	101	39.6	87	–49	6.3
	β(1 → 3)	–69	–107	50	–110	14.3	–84	51	9.0
GLYCAM	β(1 → 4)	–73	119	64	120	11.7	–74	–59	10.0
	α(1 → 4)	71	87	–54	101	26.9	76	–69	12.3
	β(1 → 3)	–76	–89	63	–109	13.6	–85	55	11.3
GROMOS	β(1 → 4)	–66	117	60^b^	119	19.6^b^	–70	–42	18.4
	α(1 → 4)	79	89	–60^b^	106	28.9^b^	101	–49	21.5
	β(1 → 3)	–63	–100	60^b^	–111	22.3^b^	–90	67	28.2

a Both the location of the energy minima and the relative energy levels with respect to the main minimum are given.

b In the absence of a local minimum corresponding to *anti*-ϕ conformation, the values given correspond to the lowest-energy value found for ϕ = ±60°.

### Coarse-Grained Model for Monte Carlo Simulations

A more sophisticated Monte Carlo model that takes into account the three-dimensional (3D) geometry of a polysaccharide chain, but still relies on input data from independent MD simulations, has also been proposed. In this section, we mainly describe the formal assumptions underlying the proposed coarse-grained Monte Carlo (CG MC) model. The relation of these assumptions to the physical properties of the studied systems, i.e., polysaccharides, is discussed in the Results and Discussion section. Our CG MC model is based on the following assumptions:

1. The model considers molecular systems with glycosidic linkages consisting of two O–C covalent bonds (i.e., β(1 → 4), α(1 → 4), β(1 → 3)) connecting two adjacent pyranose rings.
2. The model considers only unbranched homopolysaccharides containing the same type of glycosidic linkages throughout the chain.
3. The model considers configurations composed of saccharide backbone atoms, i.e., the repeating motif −C_*n*_–C_1_–O– with three atoms per monosaccharide unit in the chain. Other atoms are ignored. See Figure 2 for details.
4. The model requires input data of a structural (distances, angles, and dihedral angles) and thermodynamic nature (2D free-energy maps in the coordinates Φ vs Ψ). Φ and Ψ are the dihedral angles defined by the following atomic quadruplets: Φ = C_*n*_–C_1_–O–C_*n*_; Ψ = C_1_–O–C_*n*_–C_1_ (Figure 2). The introduction of alternative descriptors of the glycosidic linkage conformation (Φ and Ψ instead of ϕ and ψ) is necessary due to the absence of certain atoms (namely: O_5_ and C_*n*–1_) that define the “usual” ϕ and ψ glycosidic torsion angles.
5. The distances between the nearest atoms (C_*n*_–C_1_, C_1_–O, O–C*_n_*), the angles between the atomic triads (C_*n*_–C_1_–O, C_1_–O–C_*n*_, O–C_*n*_–C_1_), and the dihedral angle of the restricted rotation ring (O–C_*n*_–C_1_–O) remain unchanged during the MC simulation, and their values are determined based on the averages from the MD simulations. These average values correspond only to the ^4^*C*_1_ ring conformation and are given in Table S1. This last condition also applies to the case of the β(1 → 3)/GLYCAM system, where non-negligible ring distortions were observed in both unbiased and metadynamics MD simulations. In this particular case, the structure descriptors mentioned in this paragraph were averaged only over those configurations that exclusively exhibited the ^4^*C*_1_ ring conformers (last row in Table S1).
6. The 2D free-energy map ϕ vs ψ is known from an enhanced-sampling metadynamics MD simulation performed under conditions analogous to the unbiased MD simulations used to obtain the parameters in point 5. The main difference is that the 2D FEMs can correspond to more than one conformation of adjacent rings connected by the considered linkage.
7. The 2D energy maps Φ vs Ψ are obtained by a linear transformation of the 2D FEMs based on the usual ϕ and ψ angles, taking into account the periodicity of these two variables. The transformation data (the value of the shift of both ϕ and ψ values) are obtained based on linear regression of the Φ vs ϕ and Ψ vs ψ dependencies using data from unbiased MD simulations.
8. The initial configuration of the saccharide backbone in terms of possible conformations of the Φ and Ψ angles always corresponded to the global minimum on the 2D Φ vs Ψ FEM.
9. The Monte Carlo procedure involves random moves consisting of (1) selecting the linkage whose conformation is to be perturbed; (2) selecting a random point (Φ, Ψ) from the 2D FEM, determining the type of change in the Φ and Ψ angle values, and the energy corresponding to that change. In addition, the MC move is rejected if it results in a configuration where the smallest distance between any pair of atoms in the chain is less than 0.4 nm. This is equivalent to assigning a hard-sphere potential with a diameter of 0.4 nm to all non-neighboring atoms in the system.
10. Apart from the condition related to the introduction of a minimum distance (point 9), the only contribution to energy differences comes from changes in the values of the dihedral angles Φ and Ψ. Energies associated with the deformation of distances between atoms, angles between atomic triplets, or other than the aforementioned dihedral angles are not considered since these parameters are constant. Nonbonded interaction energies are also not considered.
11. Monte Carlo moves are accepted or rejected based on the Metropolis algorithm, i.e., the current energy of a given linkage is compared with the energy of a newly selected point on the 2D FEM, accepting the lower energy configuration and rejecting or accepting the higher-energy configuration using the Metropolis criterion.

The above Monte Carlo algorithm has been implemented in the VMD^45^ script written in the *Tcl* programming language. This allows certain functionalities of VMD to be used to construct the polysaccharide chain, perform matrix routines, and visualize the model. It should be noted that the script performs a very specific task and would need to be modified for more general use.

The output of the MC simulations performed with the above model is a trajectory of configurations, which in further stages of the study were analyzed for average structural parameters: end-to-end distance (*e*2*e*), radius of gyration (*R*_g_), and persistence length (*l*_p_). The *R*_g_ parameter was analyzed using the *gmx polystat* module, which is part of the GROMACS package. The details of the calculation of the persistence length are given in the corresponding paragraphs of the Results and Discussion section. Here, we only mention that all O atoms within the backbone associated with the CG MC model were used to define the polymeric bonds. The *l*_p_ values were calculated from the MC trajectory using handwritten scripts.

The MC simulations within the model described above concerned the homooligo- and homopolysaccharides containing the three types of glycosidic linkages, i.e., β(1 → 4), α(1 → 4), and β(1 → 3). For each type of linkage, the structural parameters and 2D FEMs calculated with three different force fields (i.e., CHARMM, GLYCAM, and GROMOS) were applied, resulting in nine MC models with unique properties. Furthermore, different chain lengths were assumed in the MC simulations in each of these cases.

## Results and Discussion

### Comparison of Force Fields

#### Free-Energy Maps

The three most popular carbohydrate-dedicated force fields are CHARMM,^14,24^ GLYCAM,^15^ and GROMOS.^16,25^ The first two are all-atom force fields, in contrast to GROMOS, which is a united-atom force field (aliphatic hydrogen atoms are not explicitly present). In addition, CHARMM and GLYCAM cover a wider range of carbohydrates, including functionalized compounds found in glycosaminoglycans.^46^ All of these force fields have been parameterized with respect to the conformation of the glycosidic linkage, although the parameterization strategies used are different.

Detailed conformational characteristics based on the analysis of ϕ vs ψ free-energy maps as well as other parameters will not be discussed, as such analyses have been described in papers on the parameterization of a given force field. In this section, we will focus mainly on the differences in the predictions of individual force fields.

**Figure 3:**
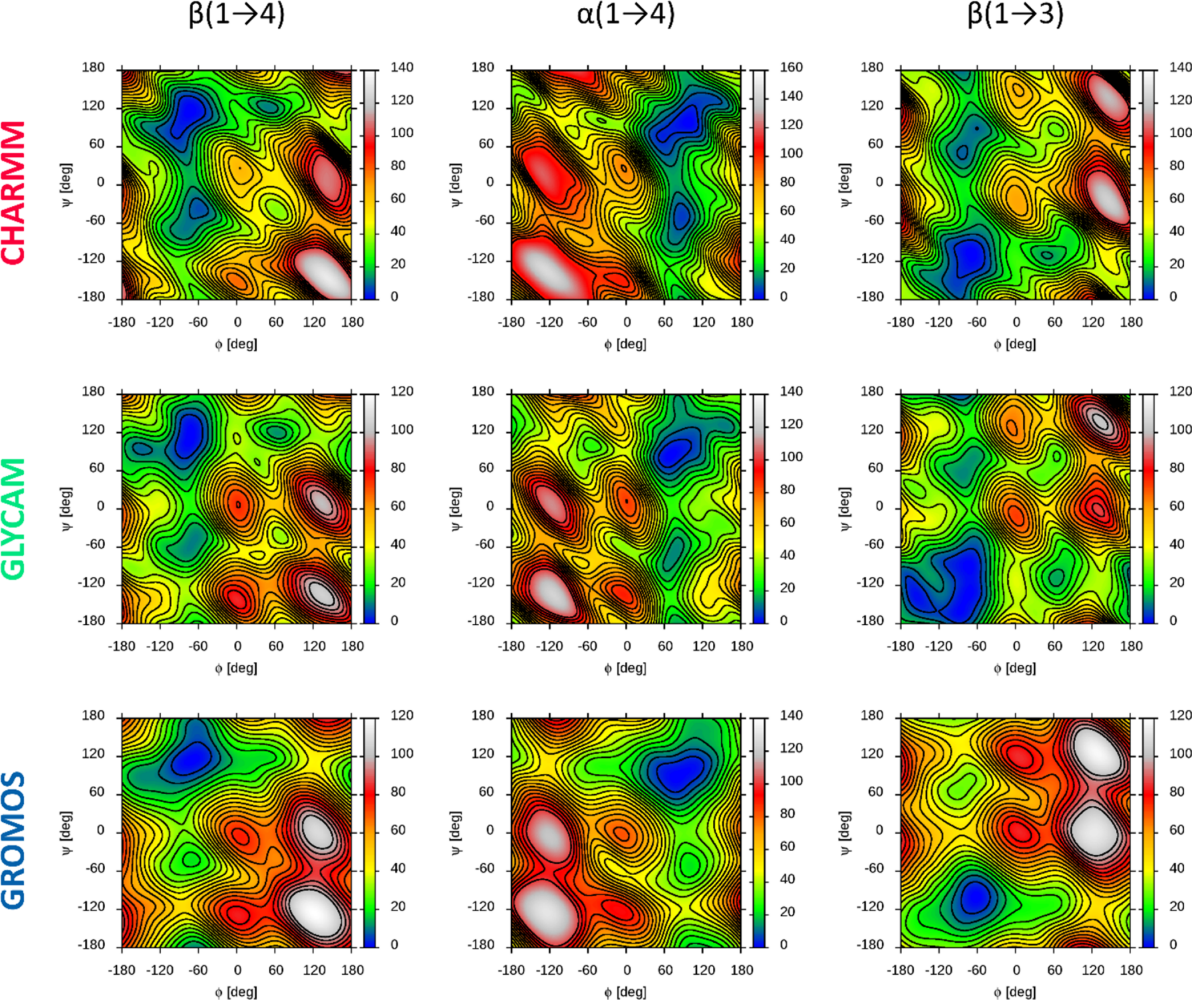
2D free-energy maps calculated for three tested force fields and the three types of glycosidic linkages connecting the glycopyranose residues. The energy scale is in [kJ/mol]. Further details are given in the text.

**Figure 4:**
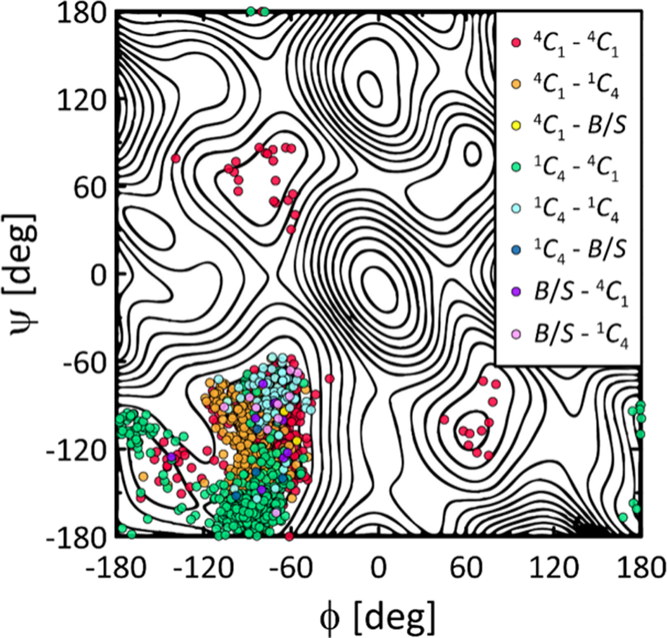
Decoupling of the contributions of the conformational states of pyranose rings adjacent to a given glycosidic linkage from the conformational states of the linkage itself. The data concern the only case where significant populations of non-^4^*C*_1_ ring conformations were found, i.e., the GLYCAM/β(1 → 3) system.

Figure 3 shows the final averaged free-energy maps for all possible linkage type/force field combinations considered in this paper. The main parameters associated with these maps are summarized in Table 1, and some of them are illustrated in Figure 4. While the qualitative character of all free-energy maps corresponding to a given linkage is similar regardless of the force field, several qualitative differences can be observed.

One of them is the predictions of the GROMOS force field with respect to the *anti*-ϕ conformation. In particular, for this force field, in contrast to CHARMM and GLYCAM, this type of reorientation is not associated with the presence of a local free-energy minimum. This does not mean that such configurations are conformationally restricted; the corresponding conformers are located on the slope of the main energy minimum, broadly stretched along the ϕ values and associated with significantly higher free-energy values. Thus, according to the predictions of the GROMOS force field, only two free-energy minima can be distinguished: the main one (*exo-syn* conformation) and that corresponding to the *anti*-ψ conformations; reorientations corresponding to the *anti*-ϕ conformation are carried out by migration of the system within the broad main minimum, without crossing the energy barrier. This observation is consistent with the free-energy maps shown in Figure 12 of ref (16), calculated with the same version of the GROMOS force field, but odd with the analogous maps shown in Figure 3 of ref (47), which were calculated with the previous version of the GROMOS force field (45a4).^48^ This may be due not only to minor differences between these two editions of the GROMOS force field but also to differences between the systems studied (both refs (16) and (47) consider disaccharides). Overall, the simpler character of the maps obtained using the GROMOS force field may be a consequence of the simplified, united-atom representation characteristic of this force field.

Another difference is the atypical nature of the main energy minimum obtained for the GLYCAM/β(1 → 3) system. This minimum occupies significantly the largest area on the ϕ vs ψ plane with respect to small energy levels (cf. Figure 3) and also has a more complicated character, consisting, in the existence, of up to three adjacent but independent minima with comparable energy levels, located in the area of −160 > ϕ > −50° and −180 < ψ < −60°. More detailed analysis revealed that these minima correspond to the superposition of several *exo-syn* conformations adopted for more than one ring shape. Both the analyses of ring shapes for the rings adjacent to the analyzed linkage based on the enhanced-sampling trajectory and independent unbiased MD simulations confirmed that the glucopyranose rings within the octamer with β(1 → 3) linkages are extremely flexible, in agreement with other studies, according to which GLYCAM predicts the highest flexibility of pyranose rings^19^ and the lowest-energy barriers between conformers of these rings.^49^ The estimated proportions of ring conformers that are part of the octamer are ^4^*C*_1_:*B*/*S*:^1^*C*_4_ = 62:11:27, where *B*/*S* is a set of boat and skew-boat conformations (estimates were based on simulations of the octamer lasting 1.1 μs). The distortion of each of the rings adjacent to the glycosidic linkage can affect its conformation, and this influence is greatest for linkages with equatorial–equatorial and axial–axial topologies,^49^ as in the present case.

Figure 4 shows the deconvolution of the contributions of individual ring shapes to the area sampled on the ϕ vs ψ map. This case shows that ring distortions can influence the increased flexibility of the glycosidic linkage. The obvious condition is that the population of alternative ring shapes must be large enough to significantly alter the landscape of the ϕ vs ψ free-energy maps. This condition is not met for any of the other systems studied here, where the populations of non-^4^*C*_1_ ring conformations are at least an order of magnitude smaller than for GLYCAM/β(1 → 3). A brief analysis of the influence of the pyranose ring shape on the conformation of the glycosidic linkage shows that deformation of the ring adjacent to the C_1_–O bond from ^4^*C*_1_ to ^1^*C*_4_ shifts the position of the energy minimum corresponding to the *exo*-*syn* conformation to lower ϕ values. The corresponding shift, calculated on the basis of the average values of ϕ and ψ angles determined for a subset of configurations with specific ring shapes, is equal to −18 °Conversely, deformation of the ring adjacent to the O–C_3_ bond leads to a slight (8°) increase in the sampled ψ values (which is particularly evident when both rings are in the ^1^*C*_4_ conformation; then, the changes reach 26°). Both trends are consistent with the results presented in ref (50) (see Figure 4 therein) based on simulations within the GROMOS force field. This indicates that the correlation between ring geometry and glycosidic linkage is consistently predicted by different force fields. The remaining qualitative data show that the most significant shifts of the average conformation of the glycosidic linkage (with respect to the case when both adjacent rings are ^4^*C*_1_) are characteristic of the following combination of ring shapes: ^1^*C*_4_–^4^*C*_1_ (shift of the average (ϕ, ψ) values by the distance of 38°), followed by *B*/*S*–^1^*C*_4_ (16°) and ^4^*C*_1_–^1^*C*_4_ (14°).

Other force fields do not predict such a high degree of pyranose ring distortion, regardless of the type of glycosidic linkage. The standard unbiased 100 ns MD simulations did not include any ring distortion events. This is consistent with quantitative data on the ring inversion energy in saccharide units that are part of an oligomer reported in refs (25,50) (in the context of the GROMOS force field) and (19) (for other force fields, including CHARMM).

In terms of quantitative differences, the main parameters concerning the location of the main energy minima and the corresponding energy levels are summarized in Table 1. All force fields predict approximately the same location of the main energy minimum, with an average difference of 13° (in terms of distance on the ϕ vs ψ plane). The most consistent predictions concern the β(1 → 4) linkage, where such a difference averages only 5°. Slightly larger differences concern the predicted location of the minima corresponding to the *anti*-ϕ conformation (15°, only for the GLYCAM vs CHARMM pair) and *anti*-ψ (18°). Moreover, in the case of *anti*-conformations, no linkage type can be distinguished as significantly better or worse in terms of agreement between force fields.

**Figure 5:**
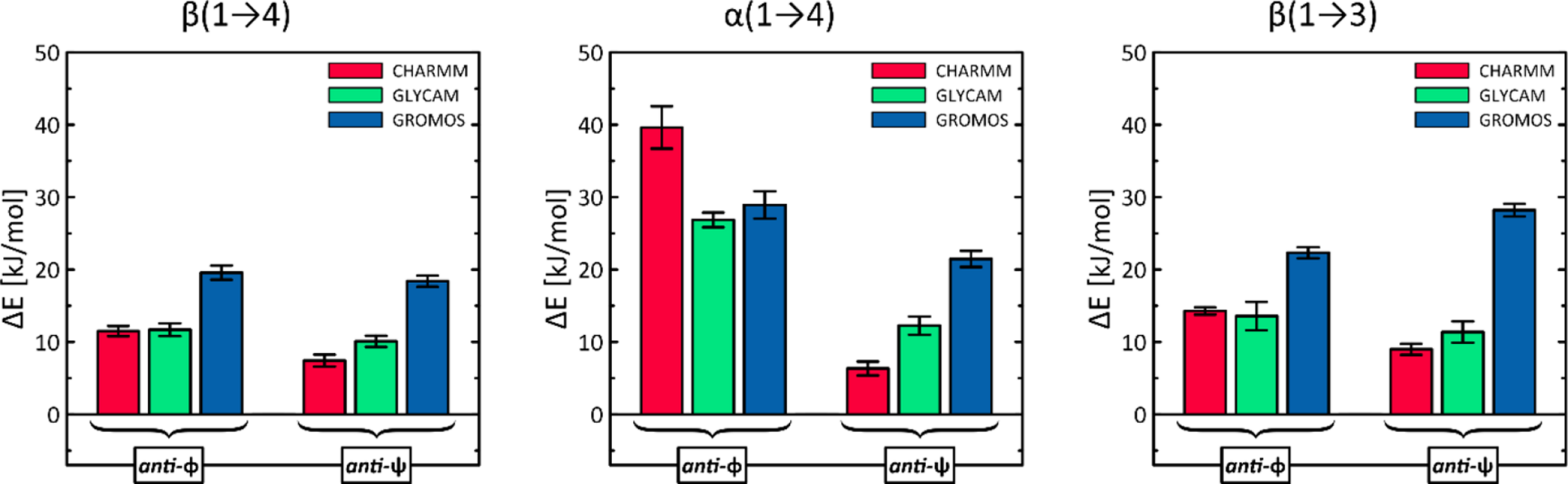
Graphical representation of the calculated energy differences between the main free-energy minimum and the minima corresponding to the *anti*-ϕ and *anti*-ψ conformations of the glycosidic linkage (see Figure 3). In the case of the GROMOS force field, the part of the data does not correspond to energy minima. The data correspond to the values given in Table 1.

More significant quantitative differences concern the predicted differences between the energies for the *exo-syn* and *anti*-ϕ as well as anti-ψ conformations. As mentioned above, in the case of the GROMOS force field, there are no separate minima at all for the *anti*-ϕ conformations, so the corresponding energy levels were estimated for the arbitrary value ϕ = −60 or 60°. The energy differences are shown in Figure 5. In all cases except the GROMOS/β(1 → 3) system, the energy level for the *anti*-ψ conformation is lower compared to that for the *anti*-ϕ reorientation, which is consistent with the influence of the *exo*-anomeric effect limiting the rotation around the ϕ angle.^51,52^ On the other hand, in the case of the GROMOS force field, where there is no separate energy minimum for the *anti*-ϕ conformers, the estimation of the corresponding energy levels is necessarily very approximate. Again, the most consistent predictions concern the β(1 → 4) linkage, where all force fields predict an energy difference between the levels of *anti*-ϕ and *anti*-ψ in the range of 1–4 kJ/mol. Similar values for other linkages differ much more: in the range of 7.5–14.5 kJ/mol (α(1 → 4)) and −6–5 (β(1 → 3)). Furthermore, there are very large qualitative differences between the energy levels for *anti*-conformations relative to the main energy minimum. For β(1 → 4) and β(1 → 3) linkages, GROMOS predicts the largest energy differences, in the range of 18–29 kJ/mol, which are significantly different from the predictions of the CHARMM and GLYCAM fields (7–14 kJ/mol). Furthermore, for the same two types of linkages, CHARMM and GLYCAM predict very similar energy levels for the *anti*-conformations, differing on average by only 3.3 kJ/mol. However, for the α(1 → 4) linkage, the predictions of all force fields are less consistent, with energy differences between *anti*-conformers ranging from 7.5 (GROMOS) to 33 kJ/mol (CHARMM). Similarly, the energy differences relative to the main minimum vary widely, especially for the *anti*-ψ conformation: from 6 (CHARMM) to 21 kJ/mol (GROMOS). On the other hand, for the *anti*-ϕ conformation, all three force fields predict very high relative energies, ranging from 27 to 40 kJ/mol.

In summary, when considering only the energy levels corresponding to *anti*-conformers, GROMOS is the force field that predicts the highest energies for glycosidic linkage reorientation. Conversely, CHARMM consistently predicts the lowest energies for reorientation to *anti*-ψ. GLYCAM predictions are intermediate but closer to those of CHARMM.

**Figure 6:**
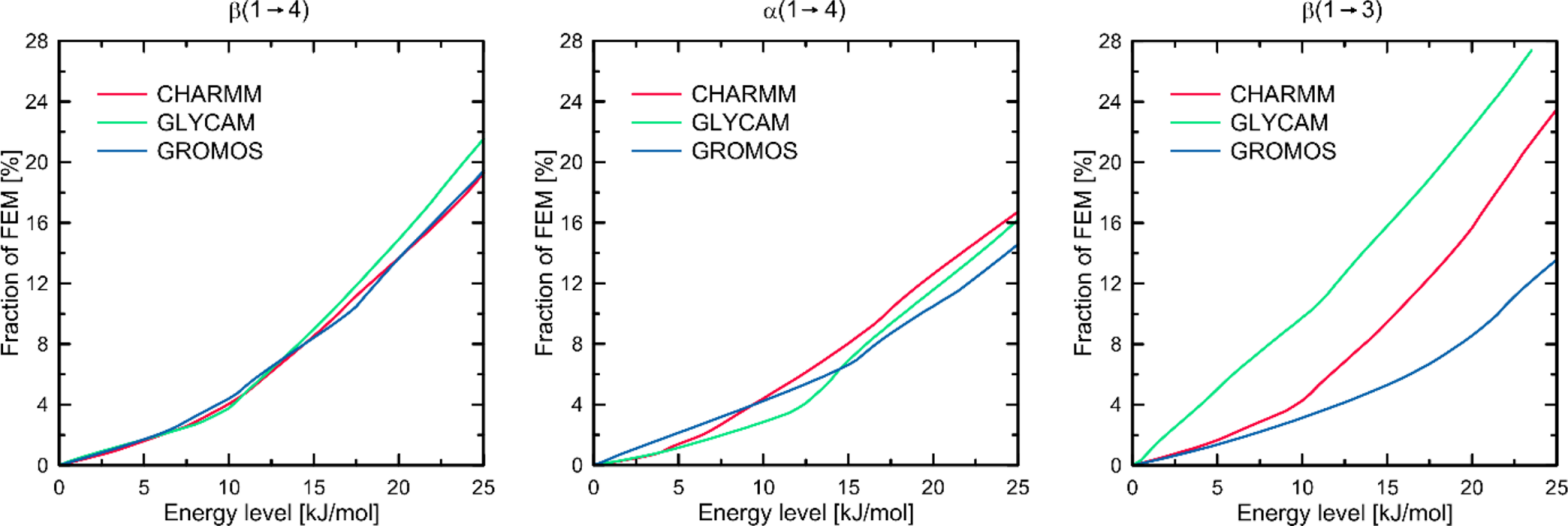
Fractions of the ϕ vs ψ free-energy maps shown in Figure 3 corresponding to energies below the given threshold.

The analysis of selected conformational states and their corresponding energies provides only partial information for estimating the flexibility of a given glycosidic linkage. In addition to well-defined conformational changes, changes in molecular geometry that are not related to leaving the main free-energy minimum are also possible. Figure 6 shows a parameter that defines the flexibility of a given linkage, i.e., the percentage of the ϕ vs ψ plane area corresponding to energy values not higher than a given level as a function of that level.

In the case of the β(1 → 4) linkage, the course of such a function is very similar for all three force fields, which is consistent with the results discussed earlier. Small differences appear only at very high (>20 kJ/mol) energy levels. The flexibility of the linkage defined in this way is predicted similarly by all force fields, whether we consider only the main energy minimum or also other regions of the free-energy map. For the α(1 → 4) linkage, differences are noticeable within the main minimum (up to 6 kJ/mol): in this region, GROMOS predicts the highest linkage flexibility, while CHARMM and GLYCAM show similar values, lower by about 50%. For higher-energy levels (>10 kJ/mol), this trend changes and CHARMM predicts greater linkage flexibility due to the availability of the lowest-energy minimum corresponding to the anti-ψ conformation. Furthermore, in the range of 4–14 kJ/mol, GLYCAM predicts the lowest linkage flexibility for α(1 → 4). Finally, the largest differences concern the β(1 → 3) linkage. As shown by the corresponding maps in Figure 3 and the previous discussion, the flexibility of this linkage predicted by GLYCAM is significantly the highest due to ring distortions and is expressed in terms of the fraction of the available free-energy map; it is 2–3 times higher than that predicted by the other force fields. This magnitude of difference is independent of the energy level. In the range of up to 10 kJ/mol, the β(1 → 3) linkage flexibilities predicted by CHARMM and GROMOS are similar. Furthermore, the trend of the flexibilities over the whole range of energy levels is as follows: GROMOS < CHARMM < GLYCAM.

#### Other Parameters and Implications for Longer Chains

The calculated conformational parameters (end-to-end distance, *e*2*e*, and radius of gyration, *R*_g_) for short octameric chains are shown in Table 2. All force fields (including the CG Martini 3 force field) predict a similar trend in *e*2*e* and *R*_g_ values, depending on the type of glycosidic linkages and changing in the order of β(1 → 4) > β(1 → 3) > α(1 → 4), assuming that we consider only configurations with dominant ring conformers, i.e., ^4^*C*_1_. This clearly indicates the most extended, rodlike conformation of the β(1 → 4)-linked octamer and a significantly more compact form of the remaining two oligosaccharides. On the other hand, it is worth noting that this trend is broken when considering the presence of non-^4^*C*_1_ ring conformers, which only occur in the GLYCAM/β(1 → 3) system. This case has already been partially discussed above.

**Table 2:** Polymer Conformation Descriptors: End-to-End Distance (*e*2*e*) and Radius of Gyration (*R*_g_)^a^

force field	linkage	*e2e* [nm]	*R*_g_ [nm]
CHARMM	β(1 → 4)	3.91 ± 0.21	1.19 ± 0.04
	α(1 → 4)	2.58 ± 0.50	0.89 ± 0.08
	β(1 → 3)	2.94 ± 0.29	0.99 ± 0.05
GLYCAM	β(1 → 4)	4.09 ± 0.15	1.24 ± 0.02
	α(1 → 4)	2.71 ± 0.67	0.94 ± 0.08
	β(1 → 3)	1.14 ± 0.94^b^	0.75 ± 0.14^b^
GROMOS	β(1 → 4)	3.90 ± 0.15	1.20 ± 0.03
	α(1 → 4)	2.84 ± 0.41	0.97 ± 0.07
	β(1 → 3)	2.97 ± 0.22	0.98 ± 0.05
MARTINI^c^	β(1 → 4)	3.70 ± 0.11	1.17 ± 0.02
	α(1 → 4)	2.45 ± 0.37	0.86 ± 0.08
	β(1 → 3)	2.96 ± 0.25	1.02 ± 0.04

a The calculations are based on unbiased MD simulations of octamers of 100 ns length (1.1 μs in the case of the β(1 → 3)/GLYCAM system). The *e*2*e* parameter depends on the C_*n*_–O distance between the nonreducing and reducing end atoms.

b The corresponding values for configurations with the pyranose ring exclusively in the ^4^*C*_1_ conformation are 2.87 ± 0.45 (*e*2*e*) and 1.02 ± 0.07 (*R*_g_).

c Definitions of *e2e* and *R*_g_ parameters refer to CG beads instead of atoms.

For the β(1 → 4) linkage, the differences between the CHARMM and GROMOS force field predictions are negligibly small, in terms of both *e*2*e* and *R*_g_. On the other hand, GLYCAM predicts the most extended conformation, consistent with *e*2*e* and *R*_g_ values that are approximately 4–5% larger than those predicted by the other two force fields.

In the case of the α(1 → 4) linkage, the differences in the force field predictions are larger. For example, the differences in *e*2*e* and *R*_g_ values between the GROMOS and CHARMM predictions are 10 and 8%, respectively. These two force fields predict the highest and lowest values of these parameters, respectively. Furthermore, the trend in *e*2*e* and *R*_g_ values changes as follows: CHARMM < GLYCAM < GROMOS.

Due to the significant contribution of non-^4^*C*_1_ ring conformations in the β(1 → 3)/GLYCAM system, it is difficult to estimate the influence of the glycosidic linkage conformation apart from the influence of ring shape changes. Considering only configurations with all rings in the ^4^*C*_1_ conformation, GLYCAM predicts the lowest *e*2*e* value among all force fields and the highest *R*_g_ value. The largest relative differences compared to the values predicted by other force fields (in particular, GROMOS) are about 4% and 5%, respectively. Furthermore, when the *R*_g_ and *e*2*e* values are determined for a long MD trajectory considering all ring shapes, they decrease to 0.75 and 1.14 nm, respectively. This clearly demonstrates the strong influence of the ring geometry on the shape of the octamer chain and shows that the direction of the corresponding change is toward more compact shapes.

Interestingly, differences in the *e*2*e* and *R*_g_ parameters estimated by individual force fields do not correlate with the level of *anti*-ϕ and *anti*-ψ conformational energies (see Figure 5). For example, consistently higher energies of *anti*-ϕ and *anti*-ψ conformers obtained for the GROMOS/β(1 → 4) system do not manifest themselves in increased *e*2*e* and *R*_g_ values for the same system. On the other hand, trends in *e*2*e* and *R*_g_ values are usually correlated, as expected from the mathematical definitions of these two quantities.

In addition, it is worth noting that all force fields predict the same trend in the range of fluctuations of the *e*2*e* and *R*_g_ parameters around the mean, which can be related to the flexibility of the chain. This trend is β(1 → 4) < β(1 → 3) < α(1 → 4), which is exactly opposite to the mean values of these parameters.

In addition, the results obtained for the carbohydrate-dedicated Martini 3 force field are consistent with the predictions of atomistic force fields, in particular, those of the CHARMM force field. This is not surprising considering that simulations within CHARMM served as a reference point during the parameterization process of this CG force field.^26^ Systematically, underestimated values of the *e*2*e* parameter are the result of a different definition, which necessarily includes limiting CG beads in the chain instead of O_1_ and O_*n*_ atoms at the reducing and nonreducing ends. This effect is much smaller for *R*_g_, where it is mitigated by averaging over a larger number of CG beads.

In summary, there are several differences between the force fields studied in terms of the predicted conformational properties of glycosidic linkages, leading to differences in the *e*2*e* and *R*_g_ parameters of up to 10%. The trends in the determined parameters depend on the type of linkage, and it is not possible to distinguish one force field as consistently predicting the “stiffest” or “most flexible” polysaccharide chains in every case. Furthermore, as seen in the example of the β(1 → 3)/GLYCAM system, the effect of differences in the predicted ring conformational equilibria by force fields can be significantly greater than differences in the conformation of the glycosidic linkage. This is of course important in systems where the non-^4^*C*_1_ conformation fraction is not negligible.

Table 3 shows the estimated populations of staggered conformers of the hydroxymethyl groups of the glucopyranose residue in a chain. Definitions are given in the footnote to Table 3. For comparison, the corresponding populations reported in the literature for glucopyranose monomers are given. The population ratios of *gg*/*gt*/*tg* are 36:58:6 (CHARMM, β-anomer),^24^ 45:51:4 (CHARMM, α-anomer),^24^ 62:36:2 (GLYCAM, α-anomer),^15^ 35:60:4 (GROMOS, β-anomer),^25^ and 38:57:5 (GROMOS, α-anomer).^25^ Overall, for monosaccharides, all force fields predict similar and high populations for the *gg* and *gt* conformers, with significantly lower populations for the *tg* conformer, indicating a relatively small influence of the anomeric configuration. Upon incorporation of the monosaccharide into the chain, the populations of the rotamers change within a rather limited range. The *tg* conformer remains the least populated of the three, with population changes in the range of 2–9%. Furthermore, all force fields consistently predict an increase in the population of the *gg* conformer at the expense of the *gt* conformer after the residue is placed in a chain with β(1 → 4) linkages. Conversely, for the α(1 → 4) linkage, CHARMM predicts a slight increase in the *gt* conformer population and a decrease in the *gg* population, GLYCAM predicts the opposite trend, while for GROMOS, the differences between monomer and chain residues are negligibly small. For the β(1 → 3) linkage, the changes in the *gg* and *gt* rotamer populations are practically negligible for the CHARMM and GROMOS cases. GLYCAM, on the other hand, shows a decrease in the *gg* rotamer population and an increase in *gt*, although the order of the populations remains unchanged when considering only configurations with all rings in the ^4^*C*_1_ geometry. In the case of a longer MD trajectory, where ring distortion has been taken into account, *gt* becomes the most populated rotamer, followed by *gg* and *tg*.

**Table 3:** Population of Staggered Rotamers of the Hydroxymethyl Group^a^

force field	linkage	*gg*	*gt*	*tg*
CHARMM	β(1 → 4)	49	42	9
	α(1 → 4)	36	62	2
	β(1 → 3)	37	58	4
GLYCAM	β(1 → 4)	72	26	2
	α(1 → 4)	74	24	2
	β(1 → 3)^b^	40	52	8
GROMOS	β(1 → 4)	62	35	3
	α(1 → 4)	37	54	9
	β(1 → 3)	37	60	3

a The populations were based on the conformation of the O_5_–C_5_–C_6_–O_6_ torsion angle, which was assigned to one of the three possible staggered conformers according to its value, i.e., *gg* (staggered conformation at −60°), *gt* (60°), and *tg* (180°).

b The corresponding values for configurations with pyranose rings exclusively in the ^4^*C*_1_ conformation are *gg*/*gt/tg* = 51:47:2.

In summary, all force fields qualitatively predict similar changes in the conformation of the hydroxymethyl group due to the placement of the glucopyranose residue in the chain, and the quantitative differences are not greater than for the simpler case of the monosaccharide.

Although hydrogen bonding cannot be considered a major determinant of the conformation of the glycosidic linkage,^49,53,54^ its presence or absence may influence the details of the conformational equilibrium to some extent. Table 4 shows the intensity of inter- and intraresidual hydrogen bonding determined from unbiased MD simulations of octamers. Due to the periodicity of the system, only two types of hydrogen bonding could be distinguished: within a residue (including the glycosidic oxygen atom) and between two adjacent residues. All force fields predict a very small contribution of intraresidue hydrogen bonding, ranging from 0 to 1.4% of the simulation frames (under the additional assumption that the considered residues adopt the ^4^C_1_ conformation). It is worth noting that GLYCAM, as the only force field, does not predict such bonds for any of the systems. Hydrogen bonds between neighboring units are much more intense and vary in the range of about 49–51, 13–42, and 15–45% for β(1 → 4), α(1 → 4), and β(1 → 3) linkages, respectively. The only case where all three force fields give the same predictions is for the system with β(1 → 4) linkages, where the difference in predicted hydrogen bond occurrences is only 2%. For the remaining systems, the force field predictions are less consistent. Both CHARMM and GROMOS predict more intense hydrogen bonding in octamers with α(1 → 4) linkages compared to those with β(1 → 3) linkages, although there are significant quantitative differences of up to 17%. On the other hand, GLYCAM predicts the opposite trend, estimating a higher intensity of hydrogen bonding in the case of octamers with β(1 → 3) linkages (45 vs 13%). Finally, considering the ring distortion in the case of the β(1 → 3)/GLYCAM system, the occurrence of both inter- and intraresidual hydrogen bonding undergoes significant changes. The occurrence of intraresidual hydrogen bonding increases to 9.5% due to the presence of nonequatorial, *syn*-axially oriented hydroxyl groups and the smaller average distance between these groups. Conversely, the incidence of inter-residual hydrogen bonding decreases to 17.5% as the distorted residue loses the ability to form stable hydrogen bonds with equatorially oriented hydroxyl groups on other residues. In summary, while the predictions regarding intraresidual hydrogen bonding are the same for all force fields, the occurrence of hydrogen bonding is predicted differently in the case of inter-residual interactions, except for the β(1 → 4)-linked saccharides.

**Table 4:** Occurrence of Hydrogen Bonding (in % of MD Simulation Time in the MD Trajectory)^a^

force field	linkage	intraresidual	inter-residual
CHARMM	β(1 → 4)	0.0	50.8
	α(1 → 4)	0.0	41.9
	β(1 → 3)	1.0	32.3
GLYCAM	β(1 → 4)	0.0	49.1
	α(1 → 4)	0.0	12.8
	β(1 → 3)	9.5^b^	17.5^b^
GROMOS	β(1 → 4)	0.1	51.1
	α(1 → 4)	0.1	36.1
	β(1 → 3)	1.4	14.7

a Occurrences calculated using the standard geometric criteria of GROMACS, i.e., a cutoff angle (hydrogen donor–acceptor) of 30° and a cutoff radius (X-acceptor) of 0.35 nm.

b The corresponding values for configurations with pyranose rings exclusively in the ^4^*C*_1_ conformation are 0.0 and 45.4.

In addition, hydrogen bonding between units separated by more than one glycosidic linkage is practically absent. In the analysis considering hydrogen bonds separated by 2 or 3 linkages, no hydrogen bonds were found regardless of the linkage topology and force field used. The only exception is the GLYCAM/β(1 → 3) system, which shows the highest flexibility of the whole molecule of the studied octamer. In this case, the occurrence of hydrogen bonding was found to be 9% for residues separated by either 2 or 3 linkages.

### Monte Carlo-Based Modeling

This section focuses on the use of two proposed Monte Carlo models: the simplified 1D model and the more complex coarse-grained (CG MC) model. Since both models are based on the results of previous MD simulations, the results presented in this section can also be interpreted in terms of similarities and differences between the force fields used for the simulations. Unless otherwise noted, all results presented are based on the more detailed Monte Carlo model, which uses the 2D FEMs and is capable of generating the 3D configurations.

#### Characteristics of the CG MC Model

The main assumptions of the proposed Monte Carlo model based on the 2D FEMs are as follows:

1. The main determinant of the conformation of a polysaccharide chain is the conformation of all glycosidic linkages within it.
2. The influence of all other degrees of freedom (orientation of ring substituents, solvent effects, inter- and intramolecular interactions including hydrogen bonds, ring shape) is either negligible or implicitly accounted for by structural parameters and 2D Φ vs Ψ free-energy maps.
3. The conformation of each glycosidic linkage in the chain is independent of the conformation of all other linkages.
4. Interactions between nonadjacent residues in the chain have a negligible effect on the conformation of the whole chain.

The above model has several limitations, some of which are general and arise directly from the assumptions made. These include

1. Consideration of only selected atoms (backbone atoms C_*n*_, C_1_, and O) out of all those belonging to the polysaccharide chain.
2. Lack of atomic details characterizing the interactions responsible for a specific conformation.
3. Neglecting all attractive and repulsive interactions within the chain and replacing them by a simple criterion of restricted distance <0.4 nm.
4. Strict dependence of the model on previous molecular dynamics (MD) simulations performed within a selected force field.
5. The inability to consider molecular systems other than a single saccharide chain of arbitrary length.
6. Lack of correlation between all degrees of freedom except the Φ and Ψ angle pair and constant values of other conformational descriptors (interatomic distances, angles, etc.).

Regarding point 2, it should be noted that the indirect characterization of the interactions causing a specific conformation can be based on the simulations mentioned in point 4. The assumption mentioned in point 3 is usually realistic due to the highly hydrophilic nature of saccharides and their well-known ability to adopt rather extended conformations. There are several examples where the conformational properties of short fragments of saccharides have been extrapolated to the case of longer chains.^55−57^ On the other hand, such extrapolation is not always justified, especially in the case of more complex systems.^58^ Assumption 6 is largely justified by the results of the MD simulation analysis, which confirm the approximately constant values of the conformational descriptors. In particular, all descriptors except angles Φ and Ψ show a narrow unimodal distribution and weak mutual correlation.

Some other limitations are mainly of a technical nature and can be easily overcome by appropriate modifications in the program implementation or by providing different input data sets from MD simulations. This group includes

1. Restriction of the implementation to the case of unbranched homopolysaccharides. Additional sets of free-energy maps combined with a more sophisticated implementation can easily allow simulations of polysaccharides containing different types of linkages and/or branched chains.
2. Limitations related to the nature of the input data. In this work, we consider only 9 individual cases (excluding changes in the length of the simulated chains), corresponding to all possible combinations of 3 force fields and 3 types of glycosidic linkages (see previous sections). Other cases, such as polysaccharides with different types of glycosidic linkages simulated within a different force field or under different simulation conditions (such as different solvents or temperatures), require only changes in structural parameters and corresponding 2D free-energy maps.

**Figure 7:**
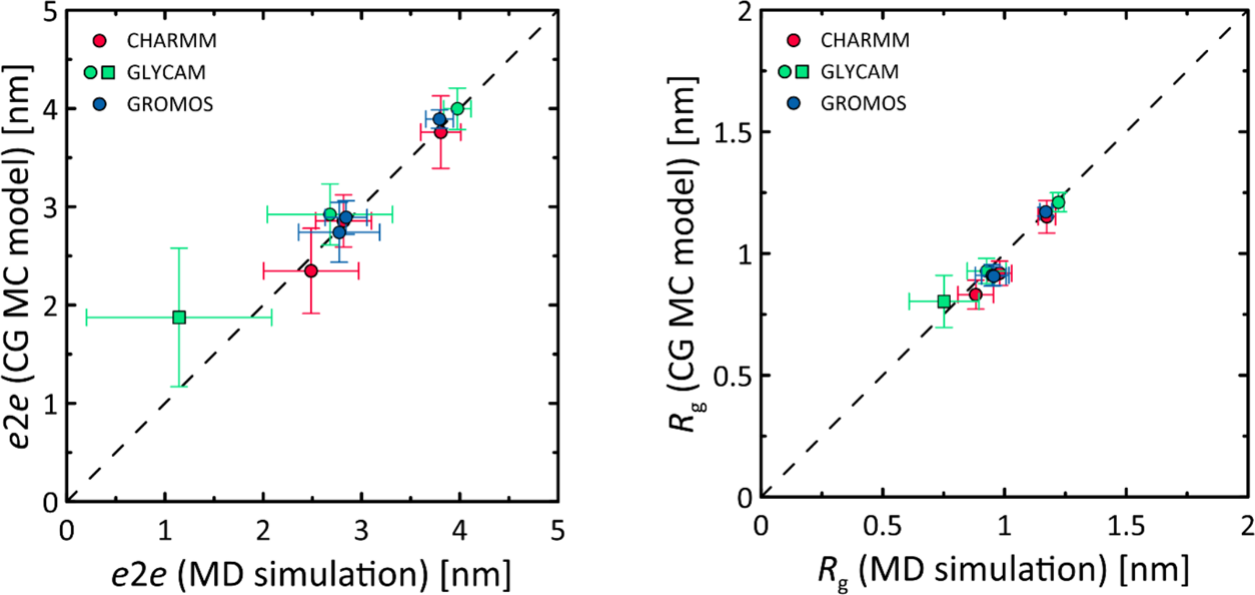
Comparison of predictions from classical force fields in MD simulations and the CG MC model. The calculations include two different parameters describing the conformation of the oligomers, namely, *e*2*e* and *R*_g_. The horizontal and vertical bars represent the standard deviations calculated from the MD and MC trajectories, respectively. The unique square symbol represents the case of the GLYCAM/β(1 → 3) system, where non-negligible ring distortion occurred and the MD-derived value corresponds to a longer, 1.1 μs long MD simulation.

Despite these limitations, the developed CG MC model has the following capabilities:

1. Simulate chains of arbitrary length.
2. Accurately predict basic parameters as a function of the conformation of the main polysaccharide chain based on MD simulation data. Figure 7 shows a comparison of predictions from the CG MC model and unbiased MD simulations for a series of octamers (these results will be discussed in the following paragraphs).
3. High computational efficiency due to a combination of factors: (a) simplification of the molecular representation; (b) absence of explicit solvent; and (c) extremely fast decorrelation of sampled configurations with respect to the number of Monte Carlo (MC) steps.
4. In line with the previous point, the computational efficiency is high enough to obtain equilibrated MC trajectories for polysaccharide chains of hundreds of residues within hours using the computing power of a desktop computer.

**Figure 8:**
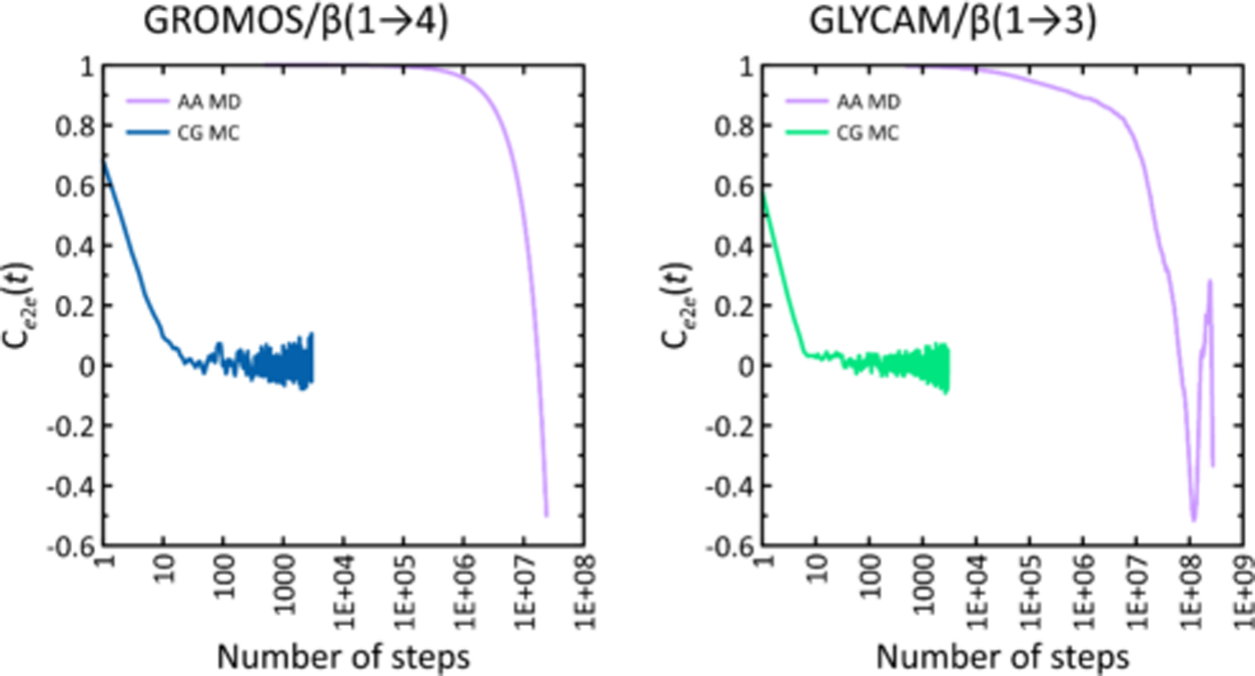
Autocorrelation functions (*C*(*t*)) calculated for the *e*2*e* parameter as a function of the number of steps (either MC or MD) for the β(1 → 3)- and β(1 → 4)-linked octamers. The data correspond to either MD or MC simulations. The MD simulations were unbiased MD simulations within the GLYCAM or GROMOS force field, whereas the MC simulations were based on the CG MC protocol (described in the Methods section) and the parameters corresponding to the given force field.

The final feature of the CG MC model is shown in Figure 8 as autocorrelation functions (*C*(*t*)) for the *e*2*e* parameter and both CG MC and MD simulations. Since the Monte Carlo simulations do not explicitly include time, the number of steps in the simulation is chosen as an independent variable. The calculations for the systems β(1 → 4)/GROMOS and β(1 → 3)/GLYCAM are shown. In the case of the second system, the most complex landscape of the free-energy map is likely to lead to the slowest decay of the corresponding *C*(*t*). The behavior of the function *C*(*t*), and in particular the rate of decrease of its value as a function of time (or in this case the number of simulation steps), shows how fast the data decorrelation progresses and allows us to estimate the time needed for the full convergence of the simulation results. Figure 8 clearly shows that the decay time of the *C*(*t*) function for Monte Carlo simulations based on the CG MC model is 7 orders of magnitude lower than the time characteristic for simulations of analogous systems using the explicit-solvent MD protocol. This is due to the aforementioned extremely rapid decorrelation of glycosidic linkage geometries caused by MC motions in configuration space.

#### Validation of the CG MC Model

Figure 7 shows a comparison of selected parameters predicted by unbiased MD simulations and the CG MC model. The comparison concerns homooctamers of glucopyranose linked by three different glycosidic linkages (β(1 → 4), α(1 → 4), and β(1 → 3)) and modeled with three different force fields (CHARMM, GLYCAM, and GROMOS) according to the methodology described in the previous sections. The predictions of the CG MC model reflect quite accurately the results based on MD simulations. The average deviation between the predicted values is 0.19 and 0.05 nm for *e*2e and *R*_g_, respectively, representing about 5% of these parameter values. A good agreement between MD simulation results and CG MC is also observed for the GLYCAM/β(1 → 3) system, where numerous ring distortions were observed in the octamer residue chains during MD simulations. This degree of freedom was not explicitly included in the CG MC model but only indirectly through φ vs ψ maps, which were then converted to Φ vs Ψ (see Figure 3). This example shows that the variable ring shape affects the structural parameters of the entire oligo/polysaccharide chain mainly by perturbing the conformational equilibrium in adjacent glycosidic linkages. Nevertheless, ignoring this most problematic system reduces the average deviation between predicted *e*2*e* values to 0.12 nm (about 4%).

Interestingly, the agreement between MD simulations and CG MC model predictions also extends to the fluctuations of the *R*_g_ and *e*2*e* parameters, expressed by their standard deviation values. In addition to their comparable magnitudes, these values are strongly correlated: *R* = 0.96 (*e*2*e*) and *R* = 0.82 (*R*_g_). This implies the significant influence of glycosidic linkage conformations on the variations of *R*_g_ and *e*2*e* parameters compared to other conformational degrees of freedom (e.g., fluctuations of valence bond angle values, bond lengths, ring shape fluctuations within the ^4^*C*_1_ conformer), which were not considered in the CG MC model.

The agreement between the predictions of the simplified CG MC model and results based on much more complex force fields primarily demonstrates the potential of the proposed model to predict the conformational properties of long polysaccharide chains. This is especially important when the property of interest is related to the structure of the large polymer, such as persistence length, radius of gyration, end-to-end distance, contour length, etc., which can be determined using coarse-grained representations.

The good agreement between CG MC model predictions and MD simulations has several implications:

1. The conformation of the polysaccharide chain is primarily determined by the glycosidic linkage conformation, and the influence of ring distortions can be implicitly accounted for by considering the linkage conformation as a result of contributions from different ring shapes.
2. Assumptions regarding the negligible influence of interactions between nonadjacent units in the chain and the lack of correlation between the conformations of different linkages in the chain are valid for saccharide conformations, at least for moderately long chains.

#### Monte Carlo-Based Modeling of Long Polysaccharide Chains

**Figure 9:**
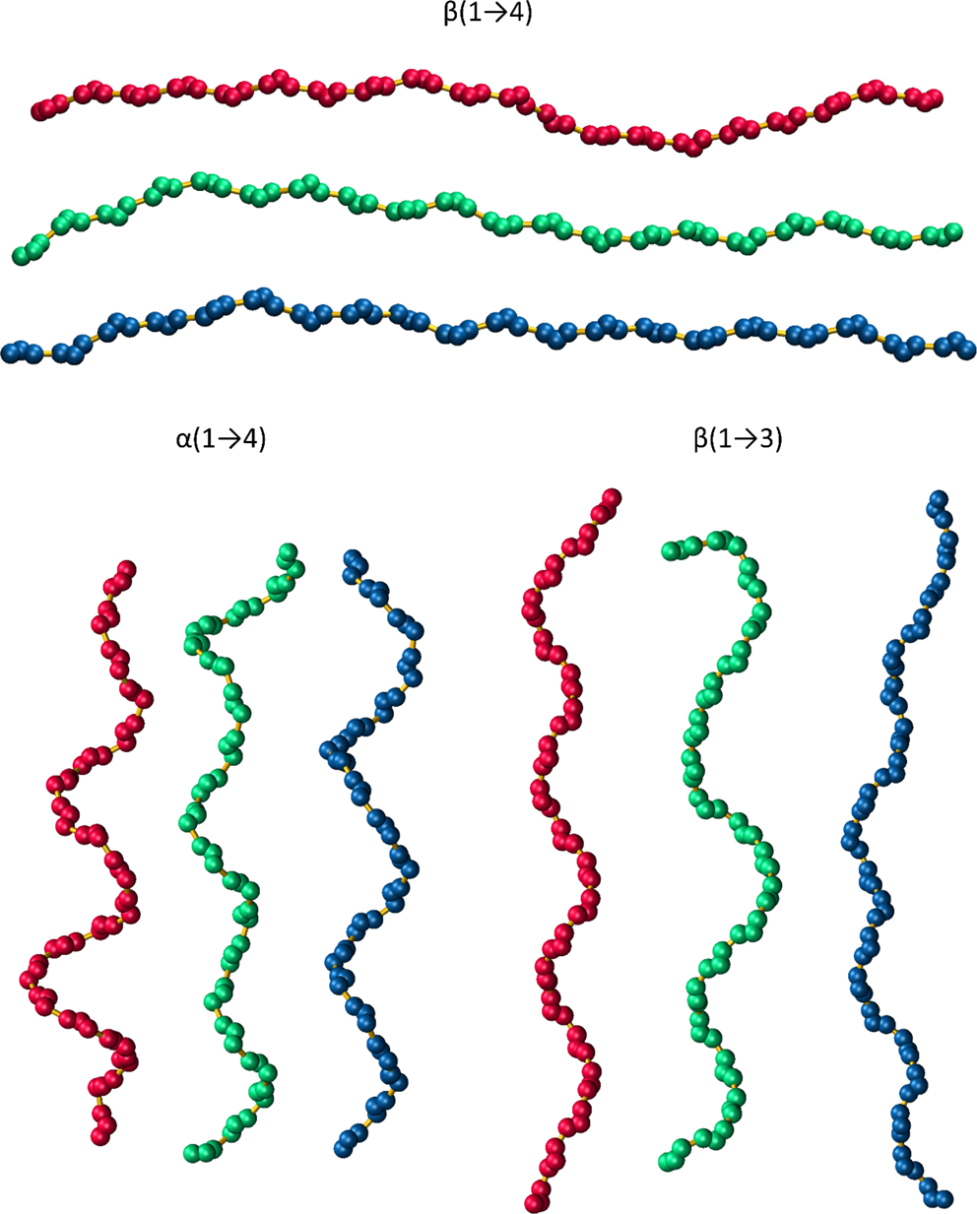
Example configurations of 20-residue polymer chains containing generated using the CG MC model. The color code is as follows: CHARMM = red, GLYCAM = green, GROMOS = blue.

The exemplary configurations of the polysaccharide chains are shown in Figure 9. Some structural features characteristic of the studied system can be observed, such as the extended shape of β(1 → 4)-linked glucan (cellulose) or helical motifs along the chains of α(1 → 4)- and β(1 → 3)-glucans (amylose and curdlan, respectively). Apart from such simple, qualitative recognition of expected structural properties, some more quantitative analyses can be performed on the data generated by applying the CG MC model. The following sections describe such analyses and their results.

**Figure 10:**
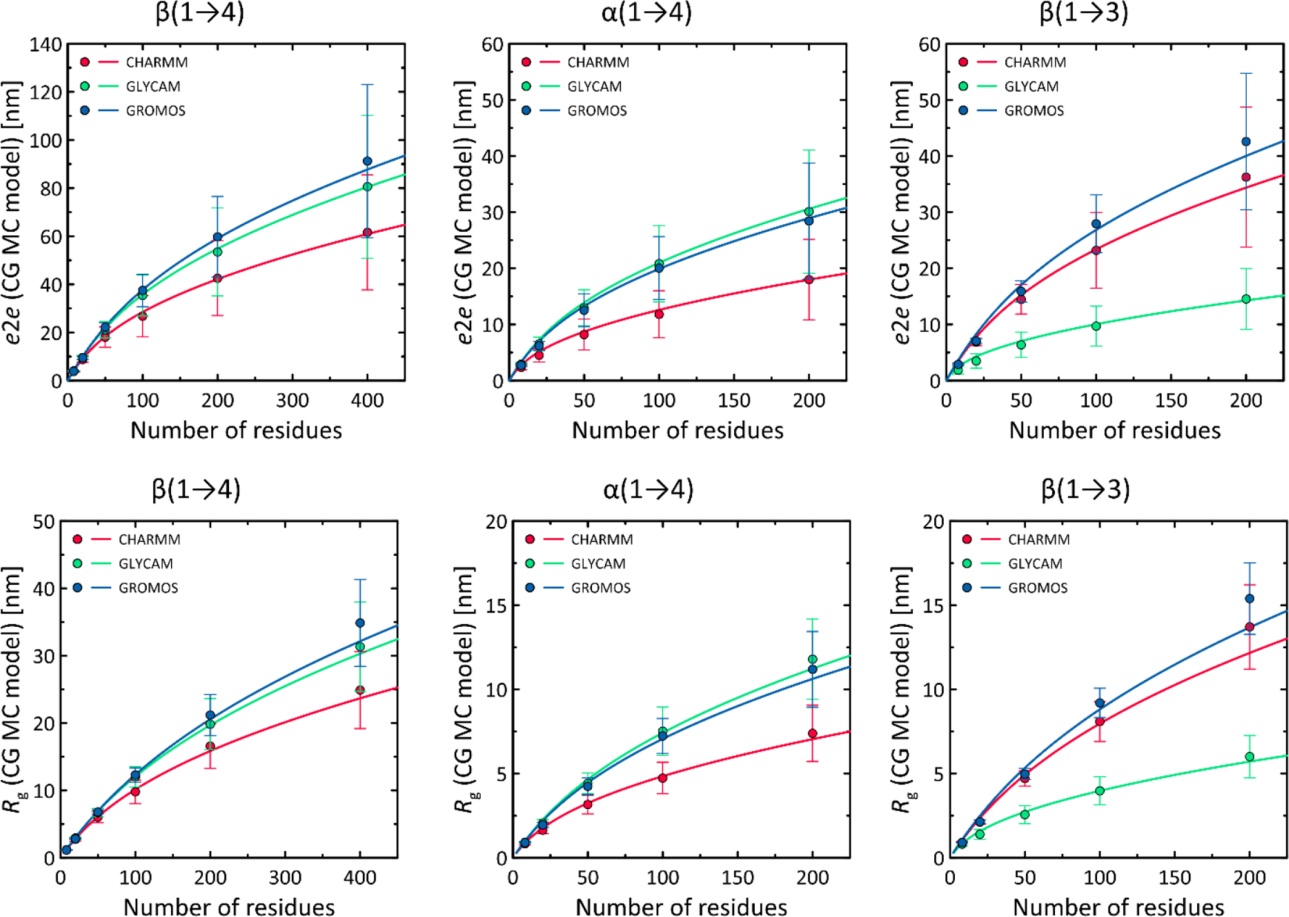
Dependence of parameters describing the conformation of polysaccharide chains (*e*2*e* and *R*_g_) on chain length (expressed in the number of monomers), calculated using the CG MC model. The solid lines are not fits but predictions based on eqs 1 and 2 with parameters determined from MD and MC simulations.

The data in Figure 10 show the relationship between *e*2*e*, *R*_g_, and the length of the chain under consideration. The dependencies shown are quite linear but only for the relatively short chains. Such a dependence is consistent with several reports in the literature^59,60^ based on MD simulations of oligo- and polysaccharides with lengths limited to 20–40 residues. However, the linearity of such a relationship breaks down with increasing chain length, and the predicted values of *e*2*e* and *R*_g_ are lower than would be expected from a simple linear dependence. A more accurate estimate is provided by relationships that relate the mean square values of *e*2*e* and *R*_g_ (denoted ⟨*e*2*e*^2^⟩ and ⟨*R*_g_^2^⟩, respectively) to the persistence length and, through the contour length parameter, to the chain length.^61^ The corresponding relations are given by eqs 1 and 2 and will be explained in the following paragraphs. Here, we only emphasize that such relations, with additional assumptions, namely, that *e*2*e* = ⟨*e*2*e*^2^⟩^1/2^ and *R*_g_ = ⟨*R*_*g*_^2^⟩^1/2^, are able to accurately capture the variation of the studied parameters as a function of the chain length. Some deviations occur only in the case of the longest chains of β(1 → 3)-glucans. More importantly, the parameters in the relevant functions (eqs 1 and 2) were not fitted as best-fit coefficients but were preset based on calculated persistence length values (see the following paragraphs). This provides clear evidence for the applicability of the Kratky–Porod worm-like chain (WLC) model^61^ to the case of glucan-based polysaccharides.

In addition to the above observation, it is worth noting significant differences between the predictions of individual force fields. In contrast to the comparison based on short octameric chains and direct MD simulations (previous section of this work), the differences in the predicted parameters are much more significant when considering longer chains composed of hundreds of residues. Excluding the most extreme case of the GLYCAM/β(1 → 3) system discussed earlier, differences approaching 50% are characteristic, for example, for the *e*2*e* parameter and the predictions of the GROMOS and CHARMM force fields in the context of β(1 → 4)-linked glucans. Even larger differences (100%) are also present for *e*2*e* determined for α(1 → 4)-linked glucans and the GLYCAM and CHARMM force fields. This analysis indicates that relatively small differences between the predictions of individual force fields considered in the context of single glycosidic linkages or short oligosaccharide chains (previous section) can accumulate and become much more significant when longer chains are considered.

While some structural parameters of the chain (including the aforementioned *R*_g_ and *e*2*e*) can be extrapolated to predict their values for chains of any length, this is not a general dependence. One parameter that is theoretically independent of chain length, and therefore a good characterization of the natural elasticity of a polysaccharide chain, is the persistence length. This value can be determined in many ways. According to the Kratky–Porod WLC model,^61^ the persistence length (*l*_p_) can be related to the value of the *e*2*e* parameter and the contour length (*l*_c_, i.e., the length of a maximally extended chain) by the following relationship
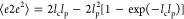
1

Benoit and Doty^62^ derived a similar relationship by linking the values of *l*_p_ and *R*_g_
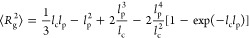
2

Additionally, one can use the autocorrelation *C*(*n*) of two bond vectors (***a***_*i*_,***a***_*i*+*n*_) separated by *n* bonds, where
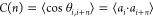
3

Then, the *l*_p_ value can be obtained by fitting the following exponentially decaying function
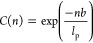
4where *b* is the average residue-to-residue bond length. The values of *C*(*n*) can be extracted from a set of molecular configurations (including, for example, the MC or MD trajectory) using eq 3.

All three methods (eq 1 applied to the average *e*2*e* value; eq 2 applied to the average *R*_g_ value; adjustment of the *l*_p_ value using eq 4 and the data generated using eq 3) were applied to input data from MC and MD simulations within the Martini 3 force field. The contour length was assumed to be equal to *b* multiplied by the number of units in the chain, while each of the bond vectors from eq 3 was defined by the coordinates of the two closest O atoms in the chain backbone (Figure 2). In the case of Martini 3, the corresponding vectors were defined by the coordinates of either B3 (β(1 → 3) linkages) or B4 (remaining linkages) beads; see the original paper for notation details. The fitting of the *C*(*n*) vs *n* data was performed in a handwritten *python* script.

Table 5 shows the values of the persistence length (*l*_p_) calculated using eqs 1–4 and the Monte Carlo or MD trajectories. While a number of quantitative differences can be observed, all methods qualitatively predict the same trends for the entire set of systems studied (for example, the correlation coefficients for the *l*_p_ values collected in columns 4–6 of Table 5 vary from 0.983 to 0.996). Therefore, the discussion of the results presented below refers collectively to all of the methods applied. It is worth noting that when eqs 3 and 4 are applied, an approximately exponential decrease in the values of the *C*(*n*) function with increasing *n* was observed for all systems, indicating the applicability of the WLC model for polysaccharides. This is in agreement with previous reports on, e.g., cellulose.^63^

**Table 5:** Average Values of *L* Parameter and Persistence Length (*l*_*p*_) Calculated Based on 1D MC and CG MC Models, Respectively^a^

force field	linkage	*L* [linkages]^b^	*l*_p_ [nm]^c^	*l*_p_ [nm]^d^	*l*_p_ [nm]^e^
CHARMM	β(1 → 4)	17.4 ± 2.4	8.57 ± 0.33	8.78 ± 0.30	8.98 ± 0.80
	α(1 → 4)	14.2 ± 2.0	1.74 ± 0.00	1.82 ± 0.01	1.47 ± 0.07
	β(1 → 3)	34.9 ± 8.3	5.47 ± 0.53	6.62 ± 0.56	7.18 ± 0.08
GLYCAM	β(1 → 4)	38.5 ± 9.6	14.56 ± 0.75	15.24 ± 0.87	16.90 ± 0.53
	α(1 → 4)	139.2 ± 50.5	4.65 ± 0.49	5.27 ± 0.56	5.62 ± 0.38
	β(1 → 3)	69.2 ± 20.2	1.04 ± 0.06	1.06 ± 0.07	0.73 ± 0.00
GROMOS	β(1 → 4)	ca. 1400 ± 680	17.45 ± 0.58	18.96 ± 0.51	22.13 ± 0.80
	α(1 → 4)	ca. 2500 ± 1400	4.16 ± 0.47	4.75 ± 0.52	5.05 ± 0.21
	β(1 → 3)	>2500	7.02 ± 1.33	8.97 ± 1.47	11.60 ± 1.10
MARTINI	β(1 → 4)				14.09 ± 0.02
	α(1 → 4)				2.28 ± 0.06
	β(1 → 3)				6.78 ± 0.04

a Calculations based on several different schemes, as indicated in further footnotes. Whenever possible, standard deviations are also given, corresponding to the variability of the variable studied. The reported *l*_p_ values correspond to the average of simulations with chains of 50 and 100 residues in length.

b From 1D MC model and chains varying in length between 500 and 5000 residues. The existence of either two (GROMOS) or three (CHARMM and GLYCAM) different conformations is assumed, corresponding to the *exo-syn* and *anti*-conformations. The energy levels are given in Table 1.

c From the WLC model and the ⟨*R*_g_⟩ value (eq 2) from MC simulations.

d From the WLC model and the ⟨*e*2*e*⟩ value (eq 1) from MC simulations.

e From eqs 3 and 4 and MC (or MD, in the case of Martini 3) simulations.

For the CHARMM and GROMOS force fields, the same trend in *l*_p_ values was observed for the three types of glycosidic linkages, i.e., β(1 → 4) > β(1 → 3) > α(1 → 4), with the intermediate *l*_p_ value observed for β(1 → 3) linkages being closer to either β(1 → 4) or α(1 → 4) for CHARMM and GROMOS, respectively. Due to the previously considered flexibility of the pyranose rings in the GLYCAM/β(1 → 3) system, a different trend was obtained for the GLYCAM force field: β(1 → 4) > α(1 → 4) > β(1 → 3).

Despite the qualitative similarities, it should be noted that the quantitative differences in the predicted *l*_p_ values are sometimes very large, depending on the applied force field. For systems with β(1 → 4) linkages (for which the predictions of all force fields discussed above are most convergent), the persistence length values range from about 8.5 nm (CHARMM) to about 16 (GLYCAM) to about 20 nm (GROMOS). For polysaccharides with α(1 → 4) linkages, the differences in predicted *l*_p_ values are smaller on an absolute scale (up to 4 nm) but significantly larger on a relative scale (almost 300% differences between CHARMM- and GLYCAM-based predictions). For this system, *l*_p_ predictions based on the GROMOS and GLYCAM force fields are quite similar (*l*_p_ around 5 nm), while those based on CHARMM estimate *l*_p_ at only 1–2 nm. A similar situation occurs for systems with β(1 → 3) linkages. GLYCAM predicts the lowest values, around 1 nm, while significantly larger values, especially in terms of relative differences, are estimated by CHARMM (around 6 nm) and GROMOS (a wider range, depending on the method, from 7 to 12 nm).

The obtained discrepancy in the results indicates the accumulation of more or less significant differences at the level of a single linkage, which become much more visible and significant when considering parameters related to the flexibility of the entire polysaccharide chain (in this case, persistence length). In addition, it is important to note the importance of both conformational changes within the main minimum on the ϕ vs ψ plane and those associated with migration to another minimum. For example, for systems with β(1 → 4) linkages that exhibit similar linkage flexibility within the main minimum (see Figure 6), the determinant of the lower *l*_p_ value for the CHARMM force field is the level of the energy minima corresponding to the *anti*-conformers (Figure 5). Conversely, despite similar energy levels for systems with β(1 → 3) linkages for CHARMM and GLYCAM, drastic differences in *l*_p_ values result from increased linkage flexibility within the main energy minimum.

The predictions of the Martini 3 force field follow the trends established by the CHARMM and GROMOS force fields (with the persistence length changing in the order: β(1 → 4) > β(1 → 3) > α(1 → 4)), with quantitative values of *l*_p_ much closer to those predicted by CHARMM. The relatively small differences between CHARMM and Martini are due to the transformation of all-atom parameters into coarse-grained parameters.

In the context of force field parameterization, such significant differences in *l*_p_ values indicate that during the parameterization process, in addition to fine-tuning parameters at the disaccharide level, it is also worthwhile to evaluate them at the level of longer chains. This mainly concerns the validation of parameters describing the flexibility of polysaccharide chains on a larger scale and their comparison with the available experimental data.

Regarding the comparison with experimental data, the estimated values of *l*_p_ for systems with β(1 → 4) linkages are close to the range of 9–12.5 nm estimated in ref^63^ based on a compilation of experimental measurements for the cellulose/water system. Interestingly, none of the results in Table 5 fall within this range; CHARMM predicts slightly lower values, while GLYCAM and Martini 3 predict slightly higher values than the limits of this range. On the other hand, the *l*_p_ values predicted by GROMOS are significantly higher. In the case of α(1 → 4)-linked glucans (amylose) in aqueous environment, the experimental values for the persistence length vary in the range of 1.09–1.52 nm,^64−66^ i.e., they are 1 order of magnitude lower than those for cellulose. Although this trend is fulfilled for all force fields, only CHARMM and Martini 3 predict persistence length values close to the experimental results; GROMOS and GLYCAM predict values 2–3 times higher. Finally, for glucans with β(1 → 3) linkage (curdlan) in aqueous solutions, experiments predict a persistence length value of about 6.8 nm,^67^ close to the values of corresponding functionalized curdlan derivatives (5.5 nm)^68^ and curdlan in DMSO (5.81 nm).^69^ In this case, the CHARMM and Martini 3 force fields give the best agreement with the experiment (Martini 3 predicts exactly the same value as the experimental one), followed by GROMOS (overestimated by about 2–4 nm). GLYCAM predicts much lower *l*_p_ values due to a significant overestimation of the ring flexibility.

Considering the general variability trend of the persistence lengths found experimentally for the three polysaccharides studied, it can be summarized that curdlan should have a persistence length 4–6 times longer than amylose, while cellulose should have a persistence length about 2 times longer than curdlan (see, e.g., Table 1 in ref (70)). This variability profile is best captured by CHARMM and Martini 3 (with the caveat of slightly overestimating or underestimating cellulose chain flexibility) and also by GROMOS (with the caveat of systematically overestimating *l*_p_ values for all systems). GLYCAM slightly overestimates the *l*_p_ value for cellulose and, to a greater extent, for amylose, but the greatest inaccuracy is associated with a significantly overestimated ring flexibility of the curdlan chain. Since this is due to the distorted conformation of the glucopyranose rings, an *ad hoc* remedy can be proposed for this system by restricting the ring conformations to the ^4^*C*_1_ shape.

Table 5 also presents the results of modeling using the simplified 1D Monte Carlo model. The main goal of creating and using this model was to investigate whether it is possible to qualitatively capture the relative stiffness of polysaccharide chains by considering only significant conformational changes (i.e., rearrangements between *exo-syn* ↔ *anti-*conformations) within glycosidic linkages. It was found that such a simplified modeling based on considering only discrete conformational states has very little correlation with the persistence length values (columns 4–6, Table 5). In addition, the parameter *L* (Table 5, column 3) varies over a wide range, from a few to several thousand residues, which in no way corresponds to realistic persistence length values. This indicates that considering only the reorientation of glycosidic linkages in discrete *exo-syn*, *anti*-ϕ, and *anti*-ψ states is not sufficient to reliably estimate the flexibility of a polysaccharide chain. In addition, it is necessary to consider the inherent flexibility associated with conformational fluctuations within the main conformation. Furthermore, the use of this 1D model demonstrates that the approximation of the conformation of a polysaccharide chain as rigid segments (corresponding to successive glycosidic linkages in the dominant *exo*-*syn* conformation) separated by kinks in the chain (*anti* conformation of the linkages) is not justified, regardless of the force field used to determine the energy levels.

## Conclusions

This work has addressed several issues related to the molecular modeling of the conformation of the glycosidic linkage, which is a fundamental determinant of the dynamic structure of carbohydrates.

In the first stage of the study, a detailed comparative analysis of three atomistic biomolecular carbohydrate-dedicated force fields (CHARMM, GLYCAM, and GROMOS) was performed with respect to their predictions concerning a number of structural and thermodynamic parameters associated with the conformation of three types of linkages between glucopyranose units: α(1 → 4), β(1 → 3), and β(1 → 4). Some aspects of the comparative analyses included also the coarse-grained, carbohydrate-dedicated Martini 3 force field. While the most important structural parameters, such as the types of main and secondary conformers on the ϕ vs ψ plane (glycosidic torsion angles), are similar for all tested force fields, a more detailed analysis revealed several qualitative and quantitative differences. Qualitative differences include the absence of separate free-energy minima for *anti*-ϕ conformations in the case of the GROMOS force field and exceptionally flexible pyranose rings in the case of the GLYCAM force field and the β(1 → 3)-linked saccharide, which significantly increases the flexibility of glycosidic linkages. In terms of quantitative differences, significant discrepancies were found in the predicted relative energy levels of *anti*-ϕ and *anti*-ψ conformers (for all types of linkages), the flexibility of glycosidic linkages (for α(1 → 4) and especially β(1 → 3) linkages), and much smaller differences in the frequency of hydrogen bonds between and within monomers in the chain, as well as the conformations of hydroxymethyl groups. These observed differences translate into variations in predicted properties related to the entire carbohydrate chain, such as radius of gyration and end-to-end distance. Differences in the average values of parameters predicted by different force fields typically fluctuate around a few percent when considering short, oligomeric chains, although they are much larger in the case of the GLYCAM/β(1 → 3) system due to ring distortions. In general, considering the entire set of parameters studied, the highest agreement between force fields occurs for systems with β(1 → 4) linkages, while the lowest agreement occurs for β(1 → 3) linkages.

In the second stage of the study, we developed a coarse-grained model (CG MC) that includes only 3 atoms per monomer and defines the backbone of the polysaccharide as a repeating −C_*n*_–O–C_1_– motif. This model is strictly based on the MD simulation data (and consequently on the force field used for it) and is designed to be used for Monte Carlo simulations. In particular, the most important type of input data for the created model is the 2D free-energy map ϕ vs ψ. The main advantage of the proposed model over traditional, explicit-solvent MD simulations lies in its computational efficiency, allowing the rapid generation of a set of configurations for carbohydrate chains of any length. In this work, we have demonstrated, among other things, the significant potential of the CG MC model to effectively determine the values of the persistence length and other polymer properties. In addition, this model was used to deepen the comparative analysis between force fields. The Monte Carlo simulation results showed that even small differences in the predicted conformational properties accumulate when the structure and flexibility of the polysaccharide chain are considered on a large scale. Consequently, the predicted values of the persistence length can differ by a factor of 2 or even more for the same system, depending on the force field. Such divergent results suggest the importance of including parameters characteristic of longer carbohydrate chains in the force field parameterization. Overall, the CHARMM and Martini 3 force field predict the persistence length values closest to the corresponding experimental data, followed by GROMOS and GLYCAM.

## Data Availability

The study was performed using open source software (GROMACS, PLUMED) or handwritten codes. The GROMACS compatible input files for MD simulations and the *Tcl* code for CG MC simulations are provided in the Supporting Information.

## Supporting Information Available

Input files for MD simulations (in GROMACS format); the parameters for CG MC simulations (structural parameters in Table S1 and Φ vs Ψ free-energy maps); and the *Tcl* code for CG MC simulations (ZIP)
