# Supplementary material for: The Conformation of Glycosidic Linkages According to Various Force Fields: Monte Carlo Modeling of Polysaccharides Based on Extrapolation of Short-Chain Properties
Source: J Chem Theory Comput. 2024 Jul 10;20(14):6350–68. doi: 10.1021/acs.jctc.4c00543 (PMC11270825; doi:10.1021/acs.jctc.4c00543)
Supplement: Supplementary file 1 — ct4c00543_si_001.zip [file ct4c00543_si_001.zip › Supporting Information.docx]

**Supporting Information**

**The Conformation of Glycosidic Linkages According to Various Force Fields. Monte Carlo Modeling of Polysaccharides Based on Extrapolation of Short Chain Properties.**

**Valery Lutsyk^1^, Pawel Wolski^1^, Wojciech Plazinski^1,2,^***

^1^ Jerzy Haber Institute of Catalysis and Surface Chemistry, Polish Academy of Sciences

Niezapominajek 8, 30-239 Krakow, Poland

^2^ Department of Biopharmacy, Medical University of Lublin

Chodzki 4a, 20-093 Lublin, Poland

* Corresponding author; e-mail: wojtek_plazinski@o2.pl

Content:

- MD_data.zip: input files for atomistic MD simulations (in GROMACS format).
- CGMC.zip: the parameters for CG MC simulations (Φ vs. Ψ free energy maps); the *Tcl* code for CG MC simulations.
- Table S1.xlsx: structural parameters from MD simulations.
