# Supplementary material for: The Conformation of Glycosidic Linkages According to Various Force Fields: Monte Carlo Modeling of Polysaccharides Based on Extrapolation of Short-Chain Properties
Source: J Chem Theory Comput. 2024 Jul 10;20(14):6350–68. doi: 10.1021/acs.jctc.4c00543 (PMC11270825; doi:10.1021/acs.jctc.4c00543)
Supplement: Supplementary file 1 — ct4c00543_si_001.zip [file ct4c00543_si_001.zip › MD_data.zip!/MD_data/gromos/1.B1-4/gromos53a6c.ff/forcefield.doc]

GROMOS96 53a6carbo force field (JCC 2011 vol 32 pag 998, doi 10.1002/jcc.21675)
